# *Tetrahymena* Glutathione Peroxidase Family: A Comparative Analysis of These Antioxidant Enzymes and Differential Gene Expression to Metals and Oxidizing Agents

**DOI:** 10.3390/microorganisms8071008

**Published:** 2020-07-05

**Authors:** Liliana L. Cubas-Gaona, Patricia de Francisco, Ana Martín-González, Juan Carlos Gutiérrez

**Affiliations:** 1 ANSES (Laboratoire de Ploufragan-Plouzané-Niort, UVIPAC), 22440 Ploufragan, France; liliana.cubas-gaona@anses.fr; 2 Department of Molecular Evolution, Centro de Astrobiología (CSIC-INTA), Carretera de Ajalvir km 4, Torrejón de Ardoz, 28850 Madrid, Spain; pdefrancisco@cab.inta-csic.es; 3 Departamento de Genética, Fisiología y Microbiología, Facultad de Biología. C/. José Antonio Nováis, 12. Universidad Complutense (UCM), 28040 Madrid, Spain; anamarti@bio.ucm.es

**Keywords:** Glutathione peroxidases, selenocysteine, SECIS elements, gene expression, oxidative stress, *Tetrahymena*

## Abstract

In the present work, an extensive analysis of the putative glutathione peroxidases (GPx) of the eukaryotic microorganism model *Tetrahymena thermophila* is carried out. A comparative analysis with GPx present in other *Tetrahymena* species and other very taxonomically diverse ciliates is also performed. A majority of ciliate GPx have replaced the selenocysteine (Sec) by Cys in its catalytic center, so they can be considered as phospholipid hydroperoxide glutathione peroxidases (PHGPx). Selenocysteine insertion sequence (SECIS) elements have been detected in several ciliate GPx that do not incorporate Sec in their amino acid sequences, and conversely, in other ciliate GPx with Sec, no SECIS elements are detected. These anomalies are analyzed and discussed. From the phylogenetic analysis using the ciliate GPx amino acid sequences, the existence of extensive intra- and interspecific gene duplications that produced multiple GPx isoforms in each species is inferred. The ancestral character of the selenoproteins is also corroborated. The analysis by qRT-PCR of six selected *T. thermophila* GPx genes has shown a quantitative differential expression between them, depending on the stressor (oxidizing agents, apoptotic inducer or metals) and the time of exposure.

## 1. Introduction

Cells consuming oxygen form reactive oxygen species (ROS), which are dangerous for the cell. In addition, various environmental stressors can also cause an increase in cellular ROS production inducing oxidative stress. To counteract the harmful effects of ROS, cells can use both non-enzymatic (vitamins, glutathione, thioredoxin and other thiol- and phenol-containing molecules) and enzymatic molecules. Within these last ones, two main families/super-families of glutathione (GSH)-dependent enzymes are involved in the cellular antioxidant response: glutathione peroxidases (GPx) and glutathione transferases (GST) [1,2]. GPx, in conjunction with superoxide dismutase (SOD) and catalase (CAT), form the SOD–CAT–GPx catalytic triad, which is presents in all prokaryotic and eukaryotic aerobic cells. In addition, almost all organisms have other thiol peroxidases, named peroxyredoxins (PRx), that catalyze the reduction of hydrogen peroxide (H_2_O_2_), alkyl hydroperoxides and peroxynitrite to less reactive products using thioredoxin (Trx) as an electron donor [3,4].

Glutathione peroxidases (GPx) (EC 1.11.1.9 and EC 1.11.1.12) form the major family of thiol antioxidant enzymes and it was the first family of selenoenzymes discovered in mammals. Selenoproteins are these proteins with one or more selenocysteine (Sec or U) residues. Selenium (Se) is an essential metalloid for the majority of living systems, being in the form of Sec when it replaces sulfur (S) in the amino acid cysteine (Cys or C). Selenoproteins are present in all three domains of the tree of life, but not in all kingdoms or species. Moreover, the selenoproteome (set of selenoproteins from a species) is very variable among eukaryotes and prokaryotes. Selenoproteins can be involved in oxidative stress protection, cellular redox balance maintaining [5] and genome stability regulation [6]. Specifically, the main catalytic function of GPx is to reduce H_2_O_2_, lipid hydroperoxides and/or other organic hydroperoxides using GSH as an electron donor. However, some GPx can accept Trx or trypanothione as reducing substrates [7,8,9]. The presence of Cys or Sec in selenoproteins is crucial to the antioxidant activity of many of these enzymes [10]. In this way, GPx may or may not contain selenocysteine (Sec), so two groups can be distinguished: with Sec (classic GPx or Sec-GPx) and without Sec (NS-GPx). Many NS-GPx do not use GSH as an electron donor but instead Trx, as in *Plasmodium falciparum*, *Saccharomyces cerevisiae*, *Drosophila melanogaster* and terrestrial plants [11]. Therefore, NS-GPx could be considered as atypical PRx, although their amino acid sequence is similar to the classical GPx.

GPx are ubiquitous enzymes, appearing in prokaryotic or eukaryotic microorganisms, invertebrates, vertebrates and plants. Likewise, these enzymes present very diverse kinetics, molecular mechanisms and structural features, showing relevant differences in efficiency and specificity [12,13]. Almost all biological groups, from bacteria to mammals and plants, present various GPx isoforms [9,14,15,16,17,18,19,20]. A total of eight GPx isoforms have been detected in mammals, based on their amino acid sequence, substrate specificity, tissue location and three-dimensional structures [13]. Experimental evidence during the last decade supports that most of these isoforms are multifunctional, playing diverse physiological roles, exhibiting different tissue-specific expression patterns and environmental stress responses [21].

Initially, the GPx catalytic center was characterized as a triad consisting of Sec (U) or Cys (C), glutamine (Q) and tryptophan (W), and later as a tetrad, adding a residue of asparagine (N). This catalytic tetrad is conserved in all characterized GPx, except in the mammalian GPx8, where the Gln residue is replaced by a serine (S) [22] and in two plant GPx, where it has been replaced by a glutamic acid (E) or by a glycine (G) [12,23]. Comparative studies of the different GPx isoforms of the three domains of living beings have shown that most of the GPx possess Cys instead of Sec in their active center [12,24,25]. Sec is encoded by the stop codon UGA, but in those organisms with selenoproteins, this codon is not recognized as a stop codon due to the presence of a RNA cis-acting stem-loop sequence motif called a selenocysteine insertion sequence (SECIS) element (about 60 b in length) [26,27]. Bacterial SECIS elements are located immediately downstream of the UGA-encoding Sec [28]. In archaea and eukaryotes, the cis-acting SECIS element is in the 3’ UTR of the mRNA, being able to recognize multiple UGA-encoding Sec within the same mRNA. As an exception, a SECIS element located in the 5’ UTR has been reported in putative selenoprotein mRNA of the methanogenic archaea *Methanococcus jannaschii* [29]. The Sec insertion machinery also needs a Sec-specific elongation factor (eEFSec), Sec-tRNA^[Ser]Sec^ and a SECIS RNA-binding protein (SECISBP2), among other factors [30].

In this research work, we analyze the GPx family from the eukaryotic microorganism *Tetrahymena thermophila*. We compare it with other ciliate GPx whose macronuclear genomes are already sequenced, including other *Tetrahymena* species. The presence of SECIS and/or Sec is also analyzed and discussed. Likewise, a quantitative analysis of gene expression (by qRT-PCR) of several *T. thermophila* GPx paralog genes is carried out under different stressful conditions (including metals).

## 2. Materials and Methods

### 2.1. Strain and Culture Conditions

The *Tetrahymena thermophila* strain used in this study was SB1969 (chx1-1/chx1-1, mpr1-1/mpr1-1; pm-S, cy-S, mtII) that was kindly supplied by Dr. E. Orías (University of California, Santa Barbara, USA). This strain was axenically grown in PP210 medium (2% w/v aqueous solution of proteose peptone (Conda Pronadisa), supplemented with 10 μM FeCl_3_ and 250 μg/mL each of streptomycin sulfate (Calbiochem, Darmstadt, Germany) and penicillin G (Sigma, Saint Louis, MO, USA), and maintained at a constant temperature of 32 ± 1 °C. Ultrapure reagent grade water was used in all experiments, obtained using a Milli-Q water purification system (Millipore, Darmstadt, Germany).

### 2.2. Stress Treatments

Prior to RNA isolation to study the gene expression patterns of selected *T. thermophila* GPx genes, ciliate cultures previously grown in PP210 medium were exposed to different stress conditions: (a) Three different metals: 44.5 μM Cd^2+^ (CdCl_2_), 315 μM Cu^2+^ (CuSO_4_ ⋅ 5H_2_O) or 965 μM Pb^2+^ [Pb(NO_3_)_2_] (all from Sigma, Saint Louis, MO, USA), 1- and 24-h treatments. All metal concentrations used were below their LD_50_ (in PP210 medium) [31,32]. (b) Three different oxidative stress inducers: 100 µM H_2_O_2_ (Panreac, Barcelona, Spain) 1 h treatment, because at longer exposures, the hydrogen peroxide decomposes to water and oxygen, 2 mM menadione (MD) (Sigma, Saint Louis, MO, USA) only for a 1 h treatment, because at higher exposure times (24 h) the death of the entire cellular population was obtained and 7.7 mM paraquat (PQ) (methyl viologen, Sigma, Saint Louis, MO, USA), 1- and 24-h treatments [33]. (c) One apoptosis inducer: 0.1 mM camptothecin (CAM) (Calbiochem, Darmstadt, Germany), 1- and 24-h treatments.

### 2.3. Total RNA Isolation and cDNA Synthesis

Exponential cell cultures (1–3 × 10^5^ cells/mL), from both controls (untreated cells) and cell populations exposed to different stressors, were harvested by centrifugation at 2000 rpm for 2 min. Total RNA was isolated using the TRIzol LS Reagent (Invitrogen, Carlsbad, CA, USA). To eliminate possible contamination with cellular DNA, samples were treated with two units of the enzyme Turbo DNase (Ambion, Carlsbad, CA, USA) for 30 min at 37 °C. Subsequently, the enzyme was inactivated by adding 1% (*v*/*v*) DNase Inactivation Reagent (Ambion, Carlsbad, CA, USA). RNA concentration and purity were determined spectrophotometrically in a NanoDrop 1000 (Thermo Scientific, Wilmington, DE, USA). The RNA integrity was analyzed by RNA electrophoresis (1.2% agarose gels). The synthesis of the cDNA first strand, from 3 µg of mRNA, was performed according to the protocol supplied in the 1st Strand cDNA Synthesis kit for RT-PCR (AMV) (Roche, Mannheim, Germany), using a reaction volume of 20 µL. The components of the reaction were: 1x reaction buffer, 5 mM MgCl_2_, 1 mM dNTPs, 1.6 µg of oligo(dT)_15_ primer, 50 units RNase inhibitor and 20 units of RT AMV reverse transcriptase.

### 2.4. Quantitative Real Time RT-PCR

Samples of cDNA were amplified and quadrupled in 96 microtiter plates (Applied Biosystems, Foster, CA, USA). Each PCR reaction (20 µL total volume) contained: 10 µL of SYBR Green PCR master mix 1x (Takara, Mountain View, CA, USA), 0.2 µM of each primer (primer sequences are showed in Table S1) and 5 µL of a 1/10 cDNA dilution. Real-time PCR reactions were carried out in an ABI PRISM 7900 HT fast real-time PCR system (Applied Biosystems, Foster, CA, USA) and the thermal cycling protocol was as follows: 10 min at 95 °C, followed by 40 cycles (15 s at 95 °C and 1 min at 50 °C). All controls were negative (no template control without cDNA and RT minus control using RNA as template). The specificity of each primer pair was tested by qPCR and a unique PCR product was obtained for all primer pairs, as determined by melting curve analysis.

To calculate the relative change in expression, we used the ∆∆Ct method [34]. Quantification was done relative to the reference genes (α-tubulin and β-actin) by subtracting the cycle threshold (Ct) of the reference gene from the Ct of the gene of interest; the resulting difference in cycle number is “∆Ct”. These obtained values, under control conditions (no stress), were subtracted from those obtained under a particular stress treatment, to give the “∆∆Ct” values. The fold induction is 2^−∆∆Ct^. This method requires that the PCR amplification efficiencies of all genes be similar and preferably at or above 90%. Amplification efficiency was measured by using 10-fold serial dilutions of a positive control PCR template and plotting Ct as a function of the log concentration of the template [34]. The slope of the resulting trend line is a linear function of the PCR efficiency. A slope of −3.32 indicates 100% amplification efficiency. The efficiency requirement was met for all the genes tested; their amplification efficiencies were greater than 90% and the correlation coefficients (R^2^) were all 99% (Table S2).

### 2.5. Bioinformatics Analysis

The nucleotide and amino acid sequences of all genes used in this work were obtained from the following databases: *Tetrahymena* genome comparative database (http://ciliate.ihb.ac.cn/tcgd/), Ciliate Genome Databases Wiki (http://ciliates.org/index.php/home/welcome) and the National Center for Biotechnology Information (NCBI) (https://www.ncbi.nlm.nih.gov/). Protein sequence accession numbers are shown in Table 1. Protein sequences were aligned using CLUSTAL Omega (1.2.4) (UniProt) or MUSCLE multiple sequence alignment, under default settings (http://www.phylogeny.fr/). Phylogenetic trees were constructed with the MUSCLE alignment, Gblocks curation, PhyML phylogeny program and TreeDyn viewer from the phylogeny.fr server (http://www.phylogeny.fr/). Percent identity matrixes were created by Clustal 2.1 (https://www.ebi.ac.uk/Tools/msa/clustalo/).

To determine the molecular mass of the different GPx amino acid sequences, the tool ProtParam from the ExPASy server (https://www.expasy.org/) was used. SECIS elements were predicted using the SECISearch3 tool [35], using Covels and/or Infernal programs, under default settings. The expression profile of *T. thermophila* GPx genes under different conditions (growing cells, starvation and conjugation) were obtained from the *Tetrahymena* Functional Genomics Database (TetraFGD) (http://tfgd.ihb.ac.cn/). Gene expression differences were tested for statistical significance by Student’s *t*-tests using the program Statgraphics Centurion XVI (16.1.15 version). The *p*-value was fixed in ≤0.05 for statistically significant induction values, with respect to the control (untreated culture).

## 3. Results

### 3.1. The GPx Family of T. thermophila Comprises 12 Isoforms

Twelve possible GPx isoforms were identified in the *T. thermophila* macronuclear genome. Table 1 compiles important information for all of them. The TtGPx amino acid sequences with the highest identity (98%) are TtGPx5 and TtGPx6, while the pairs TtGPx3–TtGPx10 and TtGPx10–TtGPx11 have the lowest identities: 11.6% and 13.6%, respectively. Most TtGPx genes do not have introns, except for TtGPx3, TtGPx 12 (both with one intron) and TtGPx10 (with two introns) (Table 1).

**Figure 1:**
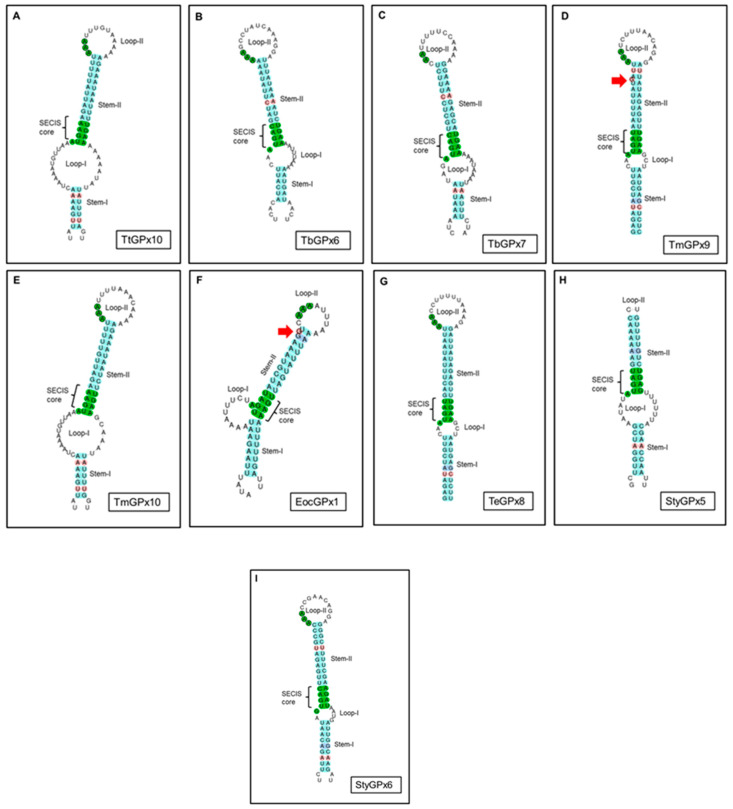
Secondary structures of the predicted selenocysteine insertion sequence (SECIS) elements of nine ciliate GPx (**A**–**I**). Canonical SECIS core and triplet of conserved adenines in loop-II are shaded in green. Stem-I and II are shaded in blue. Mismatched bases are shaded in red and/or indicated by a red arrow. Non-canonical base pairings (AG/GA) are shaded in purple. See Table 1 for species names.

Nine *T. thermophila* GPx isoforms have the conserved catalytic tetrad C/Q/W/N and only TtGPx12 has selenocysteine in it (U/Q/W/N) (Table 2). In contrast, only TtGPx10 and TtGPx11 do not have the typical catalytic tetrad. The second amino acid to the left of the catalytic tetrad is a conserved cysteine (C), sometimes substituted by a serine (S) (as in TtGPx3) (Figure S1). TtGPx12, which contains Sec in its catalytic tetrad, does not have a detectable SECIS element in its 3’ UTR, 5’ UTR or coding regions, but TtGPx10, which has neither Sec nor the typical GPx catalytic tetrad (Figure S1), has a SECIS sequence in its 3’ UTR region. The secondary structure of this SECIS element (Figure 1A) is more similar to the eukaryotic SECIS Type-I [27], presenting two main helixes or stems: I or lower (7 bp) and II or upper (11 bp), separated by one internal biggest loop or loop-I (20 b). The hairpin ends in one apical loop or loop-II (13 b). Normally, the helix-II has a conserved motif in SECIS elements, named the SECIS core (base quartet: UGAN/KGAW). In TtGPx10, this SECIS core is UGAA/UGAA (Figure 1A, Table S3). In addition, TtGPx10 shows no significant similarity to any GPx.

Ten of the twelve putative TtGPx (TtGPx1, 2, 4, 5, 6, 7, 8, 9, 11 and 12) have been identified as phospholipid hydroperoxide glutathione peroxidases (PHGPx) by the *Tetrahymena* genome comparative database. However, after using standard protein BLAST, nine of them showed their highest identity values (48–55%, and a query cover (QC) between 76 and 97%) to classical GPx from other organisms (mainly, the urochordate marine animal *Ciona intestinalis*). From them, only TtGPx1 has a 48.8% identity (QC = 95%, E (expected frequency value) = 1e^−51^) with the PHGPx6 of the angiosperm plant *Pyrus ussuriensis*.

### 3.2. A Ciliate GPx Comparative Analysis

From sequenced and available ciliate macronuclear genomes, we analyzed 91 protein sequences assigned as putative GPx. These include sequences from: (i) Four species of the genus Tetrahymena (including *T. thermophila* GPx, described in the previous section), (ii) one ciliate ectoparasite (Ichthyophthirius multifiliis) of freshwater fish and taxonomically related to Tetrahymena, (iii) one Paramecium species, (iv) two genera from the subclass Stichotrichia (Oxytricha and Stylonychia), (v) three genera from the subclass Hypotrichia (Euplotes, Monoeuplotes), (vi) one genus from the class Heterotrichea (Stentor) and vii) one pathogenic marine ciliate from the subclass Scuticociliatia (Pseudocohnilembus) (Table 1). Among the four Tetrahymena species, eight GPx isoforms were assigned in *T. borealis*, ten in *T. ellioti* and eleven in *T. malaccensis* (Table 1). The Tetrahymena-related species I. multifiliis has two GPx isoforms. The species *P. tetraurelia* presents five putative GPx isoforms. The two stichotrichous ciliates, *O. trifallax* and *S. lemnae*, have ten and nine GPx isoforms, respectively. The three hypotrichous species have two (*E. vannus*), three (*M. crassus*) and five (*E. octocarinatus*) GPx isoforms. Finally, seven putative GPx isoforms are present in both the heterotrich *S. coeruleus* and the scuticociliate *P. persalinus* (Table 1). From these data, we can infer that the average molecular mass of the ciliate GPx is around 21.7 KDa.

Among ciliates, the GPx coding genes have a very variable number of introns: From none (like the three GPx genes in *M. crassus*) to 100% of them (like the seven GPx genes from *P. persalinus*) (Table 1). *Tetrahymena* species have three or four GPx genes with introns (from one to four per gene). TeGPx9 has two gene versions for the same GPx, one with one intron and the other with two introns. Interestingly, all GPx genes from the two parasitic ciliates (*I. multifiliis* and *P. persalinus*) have introns (Table 1). StyGPx5, which is one of the largest putative GPx (122.7 KDa), has the largest number (eight) of introns. 

The presence of the catalytic tetrad C/Q/W/N is predominant in the GPx of *Tetrahymena* species: 62.5% in *T. borealis* GPx sequences, 70% in *T. ellioti*, 72% in *T. malaccensis* and 75% in *T. thermophila*. Only TtGPx12 has Sec (U) in the catalytic tetrad, the rest of the *Tetrahymena* species analyzed do not present this residue (Table 2). Two GPx sequences from each *Tetrahymena* species, except for *T. thermophila*, have a different catalytic tetrad (X/Q/W/N), in which Cys is substituted by another amino acid, represented by “X” (Table 2). The rest of the *Tetrahymena* GPx (a sequence for each species) do not have any residues of the classic catalytic tetrad (Figure S1). Only one (ImGPx1) of the two putative GPx of *I. multifiliis* has an incomplete catalytic tetrad (X/Q/W/N), and the other has no catalytic tetrad residue.

From the five putative GPx of *P. tetraurelia*, four have the catalytic tetrad C/Q/W/N, while only one (PtGPx1) has no residue of the typical tetrad (Figure S1, Table 2). The ciliates *Oxytricha* and *Stylonychia* present 40% and 33% of their GPx with the complete catalytic tetrad (C/Q/W/N), and 30 and 44% with the incomplete tetrad (X/Q/W/N), respectively. The analyzed hypotrichs, *E. vannus* and *E. octocarinatus*, have 50% and 60% of their GPx with the complete catalytic tetrad (C/Q/W/N), respectively. Similarly, *M. crassus* has the complete tetrad in all its GPx, but replaces the Cys with Sec (U/Q/W/N) (Table 2). The ciliate *Stentor coeruleus*, like *M. crassus*, is an exception among ciliate GPx, as all its GPx have Sec (U) in their catalytic tetrad (Table 1), three of them with incomplete tetrads. Similarly, *P. persalinus* presents only four GPx (57%) with incomplete catalytic tetrads (Table 2).

Many GPx in other *Tetrahymena* species (four in TbGPx, six in TeGPx and seven in TmGPx) present a conserved Cys, sometimes substituted by a Ser (TbGPx5, TeGPx7 and TmGPx8), in the second amino acid to the left of the catalytic tetrad. This Cys residue is also maintained in four GPx of *P. tetraurelia* and in SteGPx6 of *S. coeruleus*. In many other GPx from different ciliates (29.6% of the total), this Cys is replaced by Ser. Likewise, the first amino acid to the left of the catalytic tetrad may be Ser, as is the case for four GPx of *P. tetraurelia*, and Ser may even replace the Cys in the catalytic tetrad, as in OxyGPx4 (Table 2).

Within one of the highly conserved regions of many GPx (FPCNQF gray shaded section in Figure S1), there is a third or second Cys residue, present in 72.5% of the sequences, sometimes replaced by a Ser (in a 7.6% of the analyzed GPx sequences). This highly conserved Cys residue is located near the second amino acid (Q) of the catalytic tetrad (Figure S1). Furthermore, exclusively in some stichotrichous ciliate GPx, such as four GPx (44.4%) of *Oxytricha trifallax* and two GPx (22.2%) of *Stylonychia lemnae*, together with those from other organisms, a fourth non-aligned and non-conserved Cys appears, located 12 amino acids from the second catalytic tetrad amino acid (Q) (Figure S1).

### 3.3. SECIS Elements

Only nine (9.8%) of the total analyzed ciliate GPx contain SECIS elements (Table 1). Six of them (TtGPx10, TbGPx6, TbGPx7, TmGPx9, TmGPx10 and EocGPx1) are located in the 3’ UTR regions of the corresponding genes, at different distances from the stop codon (Table S3). One (TeGPx8) is located in an intron, another (StyGPx5) is within the coding region and a third (StyGPx6) is located in the 5’ UTR region (Table S3). The two StyGPx SECIS elements are located in the negative (reverse complement) DNA strand. The length of these SECIS elements is between 59 and 82 b, with an average length of ~74 b and all of them have been classified as eukaryotic SECIS Type-I. Interestingly, none of these GPx with SECIS elements has Sec in their catalytic tetrad, and, on the other hand, eleven GPx with Sec have no detectable SECIS elements (Table 1).

Figure 1 shows the secondary structures of the predicted SECIS elements of each of the nine ciliate GPx (Table 1). Table S4 shows the characteristics of these ciliate SECIS elements, from which we can highlight the following: (1) Most (77%) have a canonical SECIS core (UGAN/KGAW), where N is A, C or U, while K is U and W is A, in all cases, except in SECIS cores of StyGPx5 and 6, where W is G and K is A, respectively (Table S4). (2) Most have a triplet of conserved adenines in loop-II, except for StyGPx5, in which it is absent (Figure 1, Table S4). (3) In general, the stem-I length is smaller than that of stem-II. (4) All SECIS hairpins have mismatched bases and about half of them have non-canonical base pairings (AG/GA). (5) Most SECIS predictions are good (grade A), except for StyGPx5 and 6, which have a lower grade of prediction (grade B) (Table S3).

### 3.4. Phylogenetic Relationships

Figure S2 shows the phylogram that brings together the twelve GPx isoforms from *T. thermophila*. Two groups with a high identity are distinguished: The TtGPx7, 8 and 9 group (with an average identity of 83%) and the TtGPx1, 2, 4, 5 and 6 group (with an average identity of 86.8%). Both groups have the same amino acid pattern, including the catalytic tetrad (CXC/Q/W/N) (Figure S2). The first group is related to TtGPx12 (with an average identity of 51.8%), while the second group is associated with the TtGPx3 isoform (with a 49.8% average identity). TtGPx12 is the only *T. thermophila* GPx with Sec in its sequence (CXU/Q/W/N), and TtGPx3 also has the same catalytic tetrad as the rest (SXC/Q/W/N) but with serine (S) on the left side. TtGPx11, although somewhat connected with the rest of the canonical GPx, is separated from both groups, and TtGPx10 is completely separated or independent from the rest of the putative GPx from *T. thermophila*. These last two lack the typical GPx catalytic tetrad.

**Figure 2:**
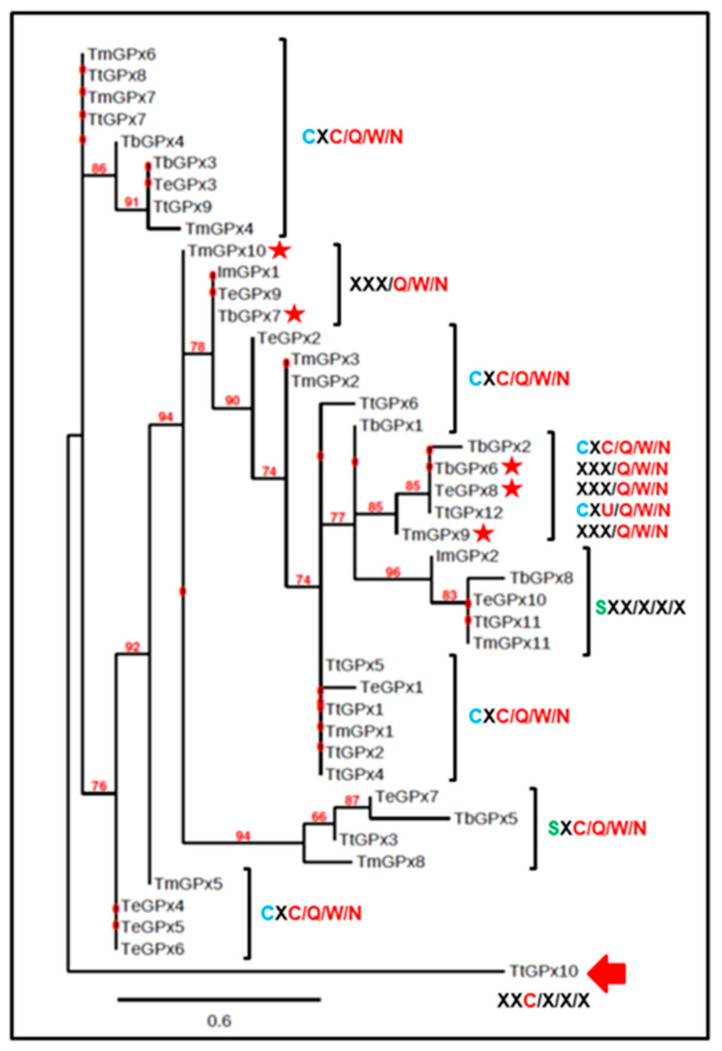
Phylogram of GPx isoforms from all selected *Tetrahymena* species. Catalytic tetrads are indicated. Species with SECIS elements are indicated by a red star. Numbers indicate bootstrap values from 2000 replicates. Branch lengths are drawn to scale, as indicated by the scale bar. See Table 1 for *Tetrahymena* species identification.

**Figure 3:**
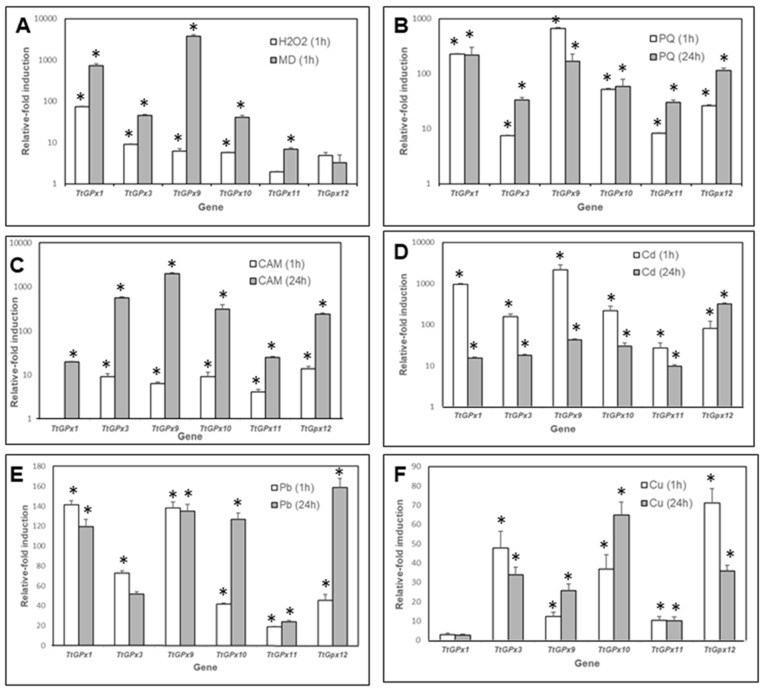
Quantitative RT-PCR results from six putative TtGPx genes, under different stressful conditions; (**A**) hydrogen peroxide or menadione (MD), (**B**) paraquat (PQ), (**C**) camptothecin (CAM), (**D**) cadmium, (**E**) lead and (**F**) copper, at 1- or 24-h treatments. Histogram bars correspond to an average value ± SD of two or three independent experiments. Asterisks indicate significant differences (*p* < 0.05), with respect to the control (untreated culture). For further explanation see the text.

The incorporation of the rest of the *Tetrahymena* species into the phylogenetic analysis gives rise to the phylogram shown in Figure 2. The GPx isoforms of the different *Tetrahymena* species appear to have a common ancestor, whereby two groups emerge. A small group consisting of nine GPx isoforms is derived from the four different species, with an average identity level of 86.6% and maintaining the typical catalytic tetrad within the pattern (CXC/Q/W/N). The second group, more numerous and complex, brings together 33 isoforms of GPx with a common origin (Figure 3), of which 16 have the pattern CXC/Q/W/N, seven contain an incomplete catalytic tetrad (XXX/Q/W/N), four show the SXC/Q/W/N pattern and another four have no catalytic tetrad, although they are connected with GPx canonicals. Here, again, the TtGPx10 isoform is completely disconnected or outside the *Tetrahymena* GPx groups. Likewise, there is a group of four isoforms (one of each *Tetrahymena* species), which do not have any residue of the characteristic GPx catalytic tetrad (SXX/X/X/X).

The incorporation of all known putative ciliate GPx amino acid sequences into the phylogenetic analysis resulted in the phylogram shown in Figure S3. The 91 ciliate GPx sequences are distributed across two large groups of 43 and 42 isoforms, along with a small group of three isoforms (PcpGPx1, 2 and 5), all with a common ancestor. Three other sequences (PcpGPx3, EocGPx3 and TtGPx10) are separated from this package. Fourteen species show no residue of the GPx catalytic tetrad (highlighted within a red square in Figure S3) and include ciliates from the *Oligohymenophorea*, *Sticotrichia* and *Hypotrichea* classes. The first large group contains GPx from ciliates located in the *Oligohymenophorea* class (Figure S3), which includes species from the subclasses: *Peniculina* (*P. tetraurelia*), *Hymenostomatida* (*I. multifiliis* and *Tetrahymena* species) and *Scuticociliatia* (*P. persalinus*). In this group, the CXC/Q/W/N pattern with the typical GPx catalytic tetrad predominates (55.8%). Other patterns bring together different species in five subgroups, for example: four isoforms of *P. tetraurelia* are in the same subgroup with the same pattern (CSC/Q/W/N), or one of each of the species of *Tetrahymena* is grouped under the same pattern (SXC/Q/W/N) (Figure S3).

The second large group brings together ciliates from the *Stichotrichia* class (*Oxytricha* and *Stylonychia*) in the same subgroup under different catalytic patterns, among which the pattern XXX/Q/W/N predominates (50%). Similarly, it brings together ciliates in other subgroups, from the *Hypotrichea* class (euplotids) and *Heterotrichea* (*Stentor*), along with a taxonomically diverse subgroup that does not have the catalytic tetrad (Figure S3).

### 3.5. TtGPx Paralog Gene Expression Patterns under Different Stressful Conditions

From the twelve paralog genes encoding GPx in *T. thermophila*, we have only been able to analyze the gene expression for six of them (*TtGPx1*, *3*, *9*, *10*, *11* and *12*), due to the high similarity of their nucleotide sequences, which makes it difficult to design specific primers that differentiate each of them. The results of the qRT-PCR analysis of cell populations subjected to different stressful conditions are shown in Figure 3.

Hydrogen peroxide, after 1 h of treatment, induces the significant over-expression of four of the six *TtGPx* genes analyzed, the *TtGPx1* gene being the most over-expressed (about 75-fold) (Figure 3A, Table S5). Menadione (MD) significantly induces the expression of five *TtGPx* genes. The highest induction values are shown by *TtGPx1* and *TtGPx9* genes (about 733- and 3800-fold, respectively) (Figure 3A, Table S5). In contrast, both *TtGPx11* and *TtGPx12* genes are not significantly induced by H_2_O_2_, nor is the *TtGPx12* gene induced by MD (Figure 3A). In Panel B of Figure 3, the results with paraquat (PQ) treatments (1 or 24 h) are shown. This herbicide induces the over-expression of all the analyzed *TtGPx* genes, especially for *TtGPx1* and *TtGPx9* and, in most cases, the 24-h treatment usually shows higher induction values.

With regard to the effect of camptothecin (CAM), all *TtGPx* genes are significantly induced except the *TtGPx1* gene after 1 h of treatment, in which there is no induction (Figure 3C). In all the analyzed genes, the induction of expression after 24 h of treatment is always higher than those obtained after 1 h of treatment. The highest induction value is shown by the *TtGPx9* gene (Figure 3C, Table S5). Likewise, cadmium (Cd^2+^) significantly induces the expression of all genes and the values obtained are higher at 1 h of treatment with respect to 24-h exposures, except for the *TtGPx12* gene, in which the opposite occurs and with the highest induction at this time (Figure 3D). At 1 h of treatment, *TtGPx1* and *TtGPx9* genes show the highest induction values, while at 24 h of treatment, it is the *TtGPx12* gene that shows the highest induction (about 318 times the control).

Lead (Pb^2+^) also induces the expression of all selected *TtGPx* genes. In some of them (*TtGPx1*, *TtGPx9* and *TtGPx11*), there is not much difference between the 1- and 24-h treatments. However, in *TtGPx10* and *TtGPx12*, there is a considerable difference between both treatments, as the 24 h treatment shows much higher induction values (Figure 3E). The highest values are shown by *TtGPx1*, *TtGPx9* (1- or 24-h treatment) and *TtGPx10* and *TtGPx12* (24-h treatment) (Table S5). Finally, copper (Cu^2+^) also induces the expression of most of the selected genes, except for *TtGPx1*, which does not present a significant induction (Figure 3F). The highest induction values are those shown by the *TtGPx12* gene (1-h treatment) and *TtGPx10* (24-h treatment) (Table S5).

## 4. Discussion

### 4.1. Ciliate GPx

Glutathione peroxidase (GPx) is a family of isoenzymes that catalyze the reduction of H_2_O_2_ or organic hydroperoxides to water or corresponding alcohol using GSH. These enzymes may or may not contain Sec (selenium-independent GPx or NS-GPx). Many NS-GPx use Trx instead of GSH as a reducing power source, and for this reason they have been considered atypical peroxyredoxins (PRx), although their amino acid sequence shows greater similarity to GPx. Among the 91 ciliate GPx isoforms analyzed, only 11 (12%) have Sec in their catalytic motif, one of them in *T. thermophila* (TtGPx12). Ciliate GPx can be considered as GPx independent of selenium, or NS-GPx, like other mammal or plant GPx, which incorporate Cys instead of Sec in the conserved catalytic motif [14,25].

PHGPx enzymes have been identified in many organisms, and in mammals, PHGPx incorporate Cys instead of Sec, and prefer Trx rather than GSH as a reducing agent [14]. PHGPx are the only GPx that use phospholipid hydroperoxides as substrates, although they can also react with hydrogen peroxide and a large number of lipid hydroperoxides (including cholesterol derivatives) [36]. The identity analysis of most ciliate GPx, regardless of whether they have Sec or Cys, shows a higher identity with PHGPx from other organisms. Even the ciliate GPx average molecular mass is around 21.7 KDa, which is similar to the molecular mass (20–22 KDa) of the PHGPx monomer protein described in mammals [25]. Although some of them have a higher identity with thioredoxin peroxidase or have a thioredoxin-like domain, such as SteGPx6 or TtGPx11, further studies are needed to determine if these ciliate GPx can interact with lipophilic substrates, such as peroxidized phospholipids, and use thioredoxin (Trx) rather than GSH as a reducing agent.

### 4.2. Presence of Sec in Ciliate GPx

Selenoproteins are present in all three domains of the tree of life (*Bacteria*, *Archaea* and *Eukarya*), however, many organisms do not use Sec, such as yeasts, higher plants and some protists (like *Cryptosporidium parvum*), because they do not have the enzymatic insertion machinery of Sec [11].

The existence of six putative selenoproteins in *T. thermophila* has been previously considered [37]: Two GPx, three thioredoxin reductases (TrxR) and one selenophosphate synthetase 2. However, our study detected only one GPx (TtGPx12) with Sec, and, assuming that the other putative selenoproteins are correct, *T. thermophila* would only have five real selenoproteins. Considering only the ciliate GPx, with the exception of *Stentor coeruleus* and *Monoeuplotes crassus*, as all their GPx are selenoproteins, the rest of the ciliate GPx (about 88%) do not have Sec in their sequences.

The Sec location in the protein sequence determined the setting of the two main groups of selenoproteins [26] in function, if Sec is closer to the C-terminus of the protein (such as TrxR) or the N-terminus (such as GPx and thioredoxin fold proteins). The analysis of the location of the Sec on the ciliate GPx shows that it is in the N-terminus of the protein. Therefore, ciliate Sec-glutathione peroxidases are classified within the second group of selenoproteins, like most of the studied GPx.

A majority (27.4%) of the ciliate GPx have a complete within the CXC/Q/W/N pattern. In second place (15.3%) are those that have a catalytic tetrad within the SXC/Q/W/N pattern and 14.2% show a XXX/Q/W/N pattern with an incomplete catalytic tetrad. Among those GPx with Sec, the SXU motif is the most abundant (72.7%). Both the SXC and SXU motifs are common in many other eukaryotic GPx [16]. In eukaryotes, Ser-tRNA^Sec^ is fundamental in the biosynthesis of Sec-tRNA^Sec^ and the incorporation of Sec into selenoproteins [38]. The incorporation of serine by a Ser-tRNA^Ser^ near Sec (SXU) or Cys (SXC) in the catalytic tetrad could be an important internal marker of the specific location where the Sec (depending on the availability of a SECIS element in the mRNA structure for translation machinery) or Cys (if there is not) should be included.

An interesting characteristic of some NS-GPx from different organisms (yeasts, fungi, plants and trypanosomatids) is the presence of a third non-aligned and non-conserved Cys located at the 8–12 amino acids of the second catalytic tetrad amino acid (Q) (Figure S1). In thioredoxin-dependent NS-GPx of *Saccharomyces cerevisiae*, *Brassica rapa* (chinese cabbage) and *Trypanosoma brucei*, the formation of an intramolecular disulfide bond appears to be an essential catalytic intermediate [18,39]. The residues involved in this bond are the conserved Cys from the catalytic tetrad and the third non-conserved Cys mentioned above. This Cys residue is essential to prevent an unspecific over-oxidation of the catalytic tetrad Cys upon reaction with a hydroperoxide substrate. Likewise, both Cys residues, together with the Gln (Q) of the catalytic tetrad, are essential for peroxidase activity [18]. In ciliate GPx, this third Cys at about 12 amino acids from residue Q and appears exclusively in some stichotrichous ciliate GPx, such as *O. trifallax* (OxyGPx3, 5, 6 and 8) and *S. lemnae* (StyGPx2 and 4). After a comparative analysis with other GPx from other organisms that also present this third non-conserved Cys, we detected that all of them have the same SXC/C/Q/C pattern, unlike other NS-GPX with a CXC/C/Q/C pattern. The SXC domain, instead of CXC, appears in all of them, but both patterns maintain three Cys residues, between two of which, a disulfide bridge could be established. Other GPx, such as CrGPx5, ScGPx1, AtGPx6 and DmGPx1 (Figure S1), like others, such as *T. brucei* III, *Leishmania mayor* I, *Oryza sativa* I and *Zea mays* GPx, also have a SXC/C/Q/C pattern. In addition, other organisms present slight modifications, such as in PfGPx1 (SXC/S/Q/C) (Figure S1) or in the cytosolic GPx1 from bovine erythrocytes, that constitutes an exception (SXU/C/Q/C), since it is a Sec-GPx [18].

Some putative ciliate GPx do not have the canonical GPx catalytic tetrad. One of each *Tetrahymena* species, with high percentages of identity between them, are within this group. A similar situation has been described in PtGPx1, PcpGPx2 and PcpGPx5, which have the highest identity with peroxyredoxins (Prx), but the analysis of their amino acid sequences showed that none of the very conserved Prx motifs [3,16] have been found. Consequently, some are similar to GPx but without the catalytic tetrad of these enzymes, and others to Prx, although they do not have the conserved motifs of the canonical Prx. However, some of them (57%), mainly among *Tetrahymena* species, have in common an SXX region instead of the CXC, SXC, CXU or SXU regions that are part of the GPx catalytic tetrad, and others (OxyGPx4 and PtGPx1) have an SXS or XXS region, respectively. We do not know what residues could act as an effective catalytic region in these enzymes, considered (by similarity) as putative ciliate GPx or Prx.

### 4.3. The Mysterious Presence or Absence of SECIS Elements in Some Ciliate GPx

One important element of the cellular machinery that facilitates the co-translational incorporation of Sec into a selenoprotein is the presence of a stem-loop in the mRNA 3’ UTR region, which is necessary to recognize the UGA codon as Sec-encoding and not a stop codon. These RNA secondary structures are known as selenocysteine insertion sequence (SECIS) elements [40,41].

Surprisingly, none of the nine ciliate GPx with SECIS elements has Sec in their sequence, and those with Sec (eleven in total) do not have a detectable SECIS element. Like in eukaryotes [42], most (66%) of the ciliate GPx SECIS elements are located in the 3’ UTR region of the mRNA, at an average distance of 76 b from the stop codon (UGA) (Table S3), which is within the distance range (50–110 b) between the SECIS element and the true stop codon (UGA) [40]. Unusually, StyGPx5 has a SECIS element in the ORF or coding region, about 337 b from the initial codon (ATG) (Table S3). The analysis of viral genomes has found that the avian fowlpox virus encodes a Sec-GPx (GPx4), and its mRNA contains a SECIS element within the coding region, and this avian GPx4 can be expressed in mammalian cells [42]. Similarly, the canarypox virus, another avian virus, also has a GPx4, but with Cys instead of Sec, and maintains the SECIS element in the coding region. The authors [42] believe that a recent mutation from Sec to Cys occurred in the canarypox virus, and the existence of a fossil SECIS element in these viruses could indicate that the selenoprotein GPx4 gene was initially acquired from the host and subsequently mutated from Sec to Cys. These same authors [42] consider that bacterial SECIS elements are in the coding region near the UGA–Sec codon because in prokaryotic organisms, transcription and translation are simultaneous processes, while the SECIS localization in the 3’ UTR of eukaryotes is an evolutionarily more recent phenomenon due to the separation of both processes (transcription in the nucleus and translation in the cytoplasm).

The SECIS location within the 5′ UTR region and intron make it more difficult to infer the evolution of these structures. In the present study, the finding of a SECIS element within the 2nd intron of the gene encoding TeGPx8, or in the 5’ UTR region of StyGPx6 (Table S3), both without Sec, leads to the idea that they have evolutionarily maintained these relics. In a selenoprotein (formate dehydrogenase) of the archaea *Methanococcus jannaschii*, a SECIS element has also been detected in the 5’ UTR region of the gene [29]. The finding of a SECIS element in a eukaryotic (ciliate) microorganism, although currently an evolutionary relic, corroborates that a SECIS element in the 5’ UTR region could also facilitate the co-translational incorporation of Sec. With respect to the presence of a SECIS element in an intron, in order for the intron to be functional, the intron should be maintained until the Sec is incorporated during translation, and then immediately removed, or a post-translational processing of the protein should be performed.

Regardless of their location, all inferred secondary SECIS RNA structures are included in Type I (Table S3), with a configuration very similar to that presented by eukaryotes [40,43]. All ciliate SECIS RNAs, except for StyGPx5 and 6 (Table S4), have a canonical SECIS core. This core is made up of four non-Watson–Crick base pairs. Within the quartet of bases, the presence of the tandem AG/GA bp constitutes a so-called kink-turn motif [44], basically identical to the eukaryotic and *Lokiarchaeota* SECIS [43]. Likewise, all ciliate SECIS RNAs, except for StyGPx5, have a triplet of adenosine in the Loop-II (or apical), which is also typical of eukaryotes. In summary, all ciliate SECIS RNAs are eukaryotic canonical SECIS elements with a good degree of prediction, except for StyGPx5 and 6, which are separated from the canonical SECIS and therefore have a lower degree of prediction (Table S3).

Usually, SECIS elements are mandatory for Sec incorporation but their presence does not necessarily lead to the incorporation of Sec. In fact, in *Drosophila* [45], a double mode of SECIS elements has been described, one leading to the incorporation of Sec and the other without the incorporation of Sec, and it is not yet known which amino acid is incorporated. All ciliate GPx with SECIS elements are not Sec-GPx, and only two of them (TtGPx10 and EocGPx1 with a 3’ UTR SECIS) have Cys in the same place that would correspond to Sec, the rest incorporate other different amino acids.

However, eleven ciliate GPx from very different taxonomic orders, with Sec in their sequences, do not present detectable SECIS elements in any region (coding or UTRs). Since a gene encoding Sec-tRNA was computationally identified in *T. thermophila* [46], we searched in the genome of *T. thermophila* and other species for the existence of some of the main elements of eukaryotic Sec insertion machinery. We found homology for this *T. thermophila* Sec-tRNA gene, which contains the anti-codon UCA that joins the UGA codon, with other putative Sec-tRNAs from other ciliate species: 96% identity in *T. borealis*, 94% in *T. elliotti*, 98% in *T. malaccesis* and 95% in *I. multifiliis*, indicating the existence of similar Sec-tRNA genes in these ciliate species. However, we have not found any other Sec-tRNA in the rest of the tested ciliates, including *S. coeruleus*, which has Sec in all its GPx.

Other elements involved in Sec biosynthesis and its co-translational incorporation into the selenoprotein, which we found in the genome of *T. thermophila*, are: (i) A selenophosphate synthetase (TTHERM_00522580), which forms monoselenophosphate, a substrate of selenocysteine synthase, with a homologous gene in *T. borealis* (EI9_12848.1). (ii) A gene encoding a selenocysteine-specific elongation factor (eEFSec) (TTHERM_00408870) involved in linking only Sec-tRNA and no other aa-tRNA [47]. (iii) A gene encoding a protein with a ribosomal L7Ae motif (TTHERM_00395870), similar to SECIS-binding protein 2 (SBP2), which is a member of the L7Ae family of RNA-binding proteins [48]. A homology analysis of this *T. thermophila* protein with SBP2 proteins from other organisms shows an identity percentage of ~22 to 25% with SBP2 from *Homo sapiens*, *Xenopus* or *Plasmodium*. The largest region of identity corresponds to the L7Ae module, which is the one interacting with the RNA SECIS core.

However, although *T. thermophila* has in its genome the main elements for the incorporation of Sec in its selenoproteins, in TtGPx12, which is a Sec-GPx, no SECIS elements are detected, after scanning with the SECISearch3 program. If this is true, and a canonical SECIS element does not exist or is not detectable using this computer tool, we do not know what the mechanism for incorporating the Sec into this selenoprotein might be. Further studies are needed to investigate the mechanism for incorporating the Sec into this selenoprotein.

### 4.4. Phylogenetic Considerations

From both GPX phylograms that include the analyzed *Tetrahymena* species and other ciliates (Figure 2 and Figure S3), we can highlight the following points:

1. It has been suggested [11,43] that Sec was probably present in the last universal common ancestor (LUCA), and has been preserved in organisms present in all three domains of the tree of life. Although ciliates also have genes encoding selenoproteins, within the genus *Tetrahymena*, it seems that the GPx family had a common ancestor which lost the Sec in its catalytic tetrad, replacing it with Cys (C/Q/W/N). A change from Cys to Sec would arise later and in a single isoform (TtGPx12) of the species *T. thermophila*. As indicated by Lobanov et al., 2007 [11], the Cys/Sec substitution can be in both directions, and both Cys → Sec or Sec → Cys changes are evolutionarily possible. When the latter occurs, the presence of a SECIS element does not have any evolutionary advantage, and tends to be lost. However, it seems that among some ciliates, this process has not happened, since supposedly the change from Sec to Cys has taken place without losing the SECIS element. Moreover, the emergence of Sec in ciliate GPx into more recent phylogenetic classes, such as *Hypotrichea* (*Monoeuplotes crassus*), could be explained as a subsequent minority acquisition among ciliate GPx, as is also the case with TtGPx12.
2. Among ciliates, including *T. thermophila*, an unusual feature is to use an alternative genetic code, in which the UAG and UAA stop codons code for glutamine, leaving only one codon (UGA) as a usable stop codon. However, *T. thermophila* presumably uses a Sec-tRNA, with a UCA anticodon, to incorporate Sec into its selenoproteins. Therefore, we can conclude that UGA is the only stop codon which is also translated to Sec, which makes *T. thermophila* the first known organism to use all 64 codons for the incorporation of amino acids in protein biosynthesis, as previously reported [49]. The fact of using a single type of stop codon (UGA), which is also used to code Sec, has probably exerted a strong selective pressure for the massive loss of this amino acid and its replacement by Cys in most ciliate GPx, despite the lower catalytic efficiency involved in replacing Sec with Cys [40].
3. The loss of the complete GPx catalytic tetrad appears to be an apomorphic character, arising from ancestors with the complete catalytic tetrad and present in one member of each studied *Tetrahymena* species and even in a related subclass (*I. multifiliis*). Similarly, the later emergence of Sec in a single *Tetrahymena* species defines it as an apomorphic character.
4. From the phylograms obtained, it can be inferred that many of the ciliate GPx isoforms, which are present in each of the species, were produced by gene duplication, as occurs in many other ciliate gene families [49,50]. The fact that similar GPx isoforms appear both within and between related ciliate species supports the idea of extensive gene duplication throughout the evolution of these microorganisms.
5. *T. thermophila*, a freshwater microorganism, has only five selenoproteins, and the rest of the ciliates probably have a similar or even lower number (with NS-GPx in almost all of them). This number of selenoproteins is closer to the seleproteomes of terrestrial organisms, such as *Dictyostelium discoideum* (with five selenoproteins), *Plasmodium falciparum* (with four) or *Drosophila melanogaster* (with three), which have smaller selenoproteomes than those living in aquatic ecosystems, such as the freshwater microalgae *Chlamydomonas reinnhardii* (with 10 selenoproteins) or the marine microalgae *Ostreococcus tauri* (with 26). Therefore, the ciliate selenoproteomes, at least the known ones, do not confirm the established hypothesis associating large selenoproteomes with aquatic life [11].

### 4.5. TtGPx Gene Expression Patterns

Table 3 shows the ranking of the relative induction expression values of selected TtGPx genes under different stress conditions. In this ranking, the genes that are most expressed (occupying 1st or 2nd place), regardless of the stressor, maintain the following order: *TtGPx9 >TtGPx1 > TtGPx12*. On the other hand, the last positions in this ranking are occupied by *TtGPx11 > TtGPx12 > TtGPx3*. The most highly expressed genes (*TtGPx9* and *TtGPx1*) have a complete GPx catalytic tetrad (CXC/Q/W/N), and the gene that is least expressed, regardless of the stressor, is *TtGPx11*, which has lost all residues of the catalytic tetrad GPx (SXX/X/X/X). The *TtGPx12* gene, the only one encoding a Sec-GPx with a complete catalytic tetrad, appears both among the three most inducible GPx genes (in 1st place five times) and also appears in the last positions (between 5th and 6th place three times in the ranking), depending on the type of stressor. The *TtGPx10* gene, which encodes a putative GPx that has only one residue (Cys) of the catalytic tetrad and which is phylogenetically outside the rest of the ciliate GPx group, is in an average position in the ranking (between 3rd and 4th place 10 times), with the exception of the Cu treatment (24 h), which is in 1st place. The different positions of these *TtGPx* genes in the ranking indicate the existence of a clear differential gene expression depending on the type of stressor.

A microarray analysis of gene expression during the *T. thermophila* life cycle [51] is available on the TetraFGD website (*Tetrahymena* Functional Genomics Database). We used this web to know the expression patterns of ten of the twelve GPx genes of this ciliate, since *TtGPx10* and *TtGPx12* are not in this database, during the growth–division cycle, starvation conditions and conjugation. According to these data, *TtGPx1* and *TtGPx11* are mostly expressed during the exponential growth phase. *TtGPx2, TtGPx5* and *TtGPx6* are mostly co-expressed between 2 and 4 h after starting conjugation, *TtGPx4* also shows a peak of expression between 2 and 6 h, and a second peak around 10 h after starting conjugation. *TtGPx7* is both over-expressed during conjugation (8–10 h of onset) and around 3 h of starvation (like *TtGPx3*). *TtGPx8* is mostly expressed at the beginning of the starvation treatment. *TtGPx9*, one of the genes that reaches higher expression values, has a maximum expression at between 15 and 24 h of starvation, although it is also expressed during the conjugation of this ciliate. These data indicate that all these GPx gene isoforms are expressed, at different levels, during different phases of the *T. thermophila* life cycle, regardless of whether their corresponding GPx enzymes have the full catalytic tetrad or not.

Among the stressors producing oxidative stress (H_2_O_2_, MD and PQ), the TtGPx9 gene shows the highest value of gene expression induction (about 3800 times the basal value) with MD. Menadione increases the levels of peroxide and superoxide radicals and is used in yeasts to experimentally induce oxidative stress by increasing the formation of superoxide ions [52]. In *S. cerevisiae*, the genes encoding GPx1-2 are also over-expressed against MD [53]. The differences in the expression levels of *TtGPx* genes, after cells are exposed to MD or H_2_O_2_, could be due to the different sensitivities of these enzymes to the stressor, or even to the different action mechanisms of these oxidizing agents. The yeast *Schizosaccharomyces pombe* also exhibits a differential response in peroxidase genes to MD or H_2_O_2_ oxidants [54]. When *Escherichia coli* is exposed to 100 µM H_2_O_2_ or 500 µM MD (for 20 min), a gene encoding a GPx, called BtuE, is expressed more (almost twice as much) in MD-treated populations than in H_2_O_2_-treated populations [55].

In general, the *TtGPx* gene expression induction induced by PQ and CAM is higher after 24 h than after 1 h of treatment. *T. thermophila* is one of the most resistant/tolerant organisms to PQ (its LC_50_ = 32.28 mM) [33]. The over-expression of the six selected *TtGPx* genes after prolonged treatment (24 h) supports the role of GPx in the antioxidant response to PQ-induced oxidative stress, as also occurs in plants and animals [56,57]. Previously, we also analyzed [33] other *T. thermophila* genes encoding antioxidant enzymes, such as thioredoxin reductase and thioredoxin peroxidase, which are also involved in antioxidant defense against this herbicide, since they were over-expressed after PQ treatment, although with less intensity than the selected *TtGPx* genes. The *TtGPx* genes were demonstrated to be sensitive even upon exposure to an apoptosis-inducing agent such as CAM, which may trigger the generation of ROS by generating stress in the endoplasmic reticulum and altering mitochondria [58]. The need for prolonged exposure (at least 24 h) to CAM to obtain a high GPx gene expression induction could be due to the need for the sufficient development of apoptosis to cause secondary oxidative stress.

With regard to metal treatments, cadmium, one of the most toxic cations, which can produce ROS and induce lipid peroxidation and oxidative stress [59], induces the highest values of gene expression for all the selected GPx genes, in both short (1 h) and longer (24 h) treatments, as in the case of the *TtGPx12* gene. There is a differential behavior between the *TtGPx12* gene and the rest of the *TtGPx* genes analyzed; most (five *TtGPx*) are highly over-expressed in the first hour of Cd-exposure, while *TtGPx12* is considerably more induced after 24 h of exposure. The fundamental difference between TtGPx12 and the rest of the TtGPx is the presence of Sec in its molecule. Cd probably has a higher affinity for the -SH groups from Cys residues, while it may have less affinity for the -SeH from Sec. This could explain the rapid response of genes encoding NS-GPx against those encoding Sec-GPx, since more of the NS-GPx would be required when their catalytic tetrad (with Cys) are blocked. In both animals [60] and plants [61], short (1 h) treatments with Cd induce an initial increase in GPx activity or an increase in the expression of GPx genes, which decreases after prolonged treatments (24 h). These data suggest that antioxidant enzymes (with -SH groups in their catalytic center) are easily blocked by Cd, so it is required to overexpress their genes to produce a greater amount of enzyme to neutralize the blocking effect. Other authors [62] suggest that these enzymes constitute a first defense line against Cd, even before inducing the synthesis of Cd-metallothioneins.

Oxidative stress plays an important role in Pb toxicity, since this metal causes a decrease in proteins with a protective function against oxidative damage and the production of ROS [63], and above all, an increase in the level of lipid peroxidation [64]. It has been proven that the binding of Pb to human GPx1 causes a decrease in the activity of this enzyme in brain tumors [63]. Similarly, experiments carried out in mouse brains with treatments for eight weeks with lead acetate increase the expression of the gene encoding the enzyme PHGPx [65]. In *T. thermophila*, all selected GPx genes are significantly induced under Pb treatment. Three of them (*TtGPx1, 3* and *9*) are induced at higher levels after 1 h of exposure, while the other three (*TtGPx10, 11* and *12*) are induced at higher levels after 24 h of exposure. The difference between the first three and the last three is the presence of Cys in the catalytic tetrad versus the presence of Sec or the absence of a complete catalytic tetrad. This confirms what could also be inferred from the response to Cd.

Copper is an essential metal, which plays a vital role as a catalytic co-factor in a variety of enzymes, but, depending on the concentration, it can behave as a toxic agent, causing ROS [66]. When there is an excess of Cu, the expression of genes involved in the antioxidant defense, such as GPx, among others, can be induced. In the algae *Scenedesmus bijugatus*, the enzyme activities of GST, SOD and GPx increase after exposure to Cu, up to double in the case of GPx [67]. Unlike Cd, and similar to Pb, it is the *TtGPx12* gene that reaches higher induction values after exposure to Cu (1 h). The *TtGPx10* gene also presents high induction values after 24 h of Cu treatment.

## 5. Conclusions

From this study, we draw the following main conclusions:

1. Ciliated protozoa GPx are mostly NS-GPx (with Cys in their catalytic tetrads), and a minority are of the Sec-GPx type.
2. Many ciliate GPx could be considered as PHGPx, because they have Cys instead of Sec as part of their catalytic center, and probably use Trx rather than GSH as a reducing substrate.
3. None of the nine ciliate GPx, where SECIS elements are detected, has Sec in their sequence, so they could be considered evolutionary relics that have remained in the genome after losing Sec or replacing it with Cys.
4. Although *T. thermophila* has in its genome the main elements for the incorporation of Sec into its selenoproteins, in the isoform TtGPx12, which is a Sec-GPx, no SECIS elements are detected, after scanning with the SECISearch3 program.
5. The loss of the complete GPx catalytic tetrad appears to be an apomorphic character, arising from ancestors with the complete catalytic tetrad, and is present in one member of each studied *Tetrahymena* species and even in a related subclass.
6. Extensive genetic duplication appears to have occurred throughout the evolution of ciliate GPx, as similar paralogs exist both within and between related ciliate species.
7. The size of the *T. thermophila* selenoproteome, and probably those from other ciliates, is closer to those present in terrestrial organisms, despite the fact that this ciliate has an aquatic life.
8. Differential gene expression patterns exist between all selected *T. thermophila* GPx genes, depending on the nature of the stressor and the time of exposure. Regarding the metal treatments, both Cd and Pb could have a greater affinity for the -SH groups of the Cys residues than for the -SeH groups of the Sec, which could explain the faster response of NS-GPx-coding genes with respect to those coding Sec-GPx.

These studies are not only relevant to better understanding this group of antioxidant enzymes and their diversity as selenoproteins, but also to understanding the functional diversity of different paralogs present in the same organism under stressful conditions. Likewise, from these analyses, we could select genes that could be useful as biomarkers of the presence of certain organic or inorganic contaminants.

## Figures and Tables

**Table 1:** Putative glutathione peroxidases (GPx) from Different *Tetrahymena* Species, Other Ciliates and Some Other Selected Organisms.

CILIATES					
Name	AccessionNumber ^(1)^	MW (KDa)	Introns	SECIS	Sec
TtGPx1	TTHERM_00630010	19.9	0	-	-
TtGPx2	TTHERM_00895660	24.5	0	-	-
TtGPx3	TTHERM_01099010	15.5	1	-	-
TtGPx4	TTHERM_00895640	20.0	0	-	-
TtGPx5	TTHERM_00895650	20.0	0	-	-
TtGPx6	TTHERM_00895630	20.0	0	-	-
TtGPx7	TTHERM_00046110	21.9	0	-	-
TtGPx8	TTHERM_00046090	22.0	0	-	-
TtGPx9	TTHERM_00141170	24.5	0	-	-
TtGPx10	TTHERM_000141169	15.5	2	+	-
TtGPx11	TTHERM_00661720	21.0	0	-	-
TtGPx12	TTHERM_000279829	20.9	1	-	+
TbGPx1	E19_04307.1	20.1	0	-	-
TbGPx2	EI9_08583.1	21.2	1	-	-
TbGPx3	EI9_04856.1	21.6	0	-	-
TbGPx4	EI9_04855.1	21.5	0	-	-
TbGPx5	EI9_19376.1	19.8	1	-	-
TbGPx6	EI9_08932.1	18.4	1	+	-
TbGPx7	EI9_04857.1	19.6	3	+	-
TbGPx8	EI9_17207.1	21.2	0	-	-
TeGPx1	E17_10142.1	19.9	0	-	-
TeGPx2	E17_10229.1	20.0	0	-	-
TeGPx3	E17_10737.1	21.4	0	-	-
TeGPx4	E17_05831.1	22.1	0	-	-
TeGPx5	E17_05830.1	22.0	0	-	-
TeGPx6	E17_05832.1	22.1	0	-	-
TeGPx7	E17_20047.1	19.4	1	-	-
TeGPx8	E17_12195.1	25.5	4	+	-
TeGPx9 ^(2)^	E17_10736.1		1	-	-
	E17_10736.2	16.0	2		
TeGPx10	E17_11408.1	21.0	0	-	-
TmGPx1	EIA_10427.1	19.9	0	-	-
TmGPx2	EIA_05417.1	19.9	0	-	-
TmGPx3	EIA_05416.1	21.8	1	-	-
TmGPx4	EIA_04640.1	21.5	0	-	-
TmGPx5	EIA_01773.1	22.1	0	-	-
TmGPx6	EIA_01775.1	22.0	0	-	-
TmGPx7	EIA_01770.1	22.0	0	-	-
TmGPx8	EIA_20673.1	19.4	1	-	-
TmGPx9	EIA_08958.1	17.1	1	+	-
TmGPx10	EIA_04639.1	19.7	3	+	-
TmGPx11	EIA_07345.1	21.0	0	-	-
ImGPx1	IMG5_104650.1	16.3	2	-	-
ImGPx2	IMG5_178250.1	24.7	1	-	-
PtGPx1	GSPATT00033641001	18.3	0	-	-
PtGPx2	GSPATT00011474001	20.8	2	-	-
PtGPx3	GSPATT00004297001	20.7	2	-	-
PtGPx4	GSPATT00002394001	21.3	2	-	-
PtGPx5	GSPATT00011189001	20.8	2	-	-
OxyGPx1	OXYTRI_14679	24.4	3	-	-
OxyGPx2	OXYTRI_14831	13.8	0	-	-
OxyGPx3	OXYTRI_00243	19.7	0	-	-
OxyGPx4	OXYTRI_11899	21.0	0	-	-
OxyGPx5	OXYTRI_09235	37.2	0	-	-
OxyGPx6	OXYTRI_00914	20.8	0	-	-
OxyGPx7	OXYTRI_24397	19.9	1	-	-
OxyGPx8	OXYTRI_17994	19.3	0	-	-
OxyGPx9	OXYTRI_19181	20.2	1	-	-
OxyGPx10	Contig6997.0.g51	25.9	2	-	-
StyGPx1	Contig8855.g9459	15.5	0	-	-
StyGPx2	Contig13525.g14435	19.0	0	-	-
StyGPx3	Contig14711.g15669	23.3	0	-	-
StyGPx4	Contig17005.g18115	19.0	0	-	-
StyGPx5	Contig18173.g19318	122.7	8	+	-
StyGPx6	Contig18431.g19575	19.8	1	+	-
StyGPx7	Contig18781.g19927	17.6	1	-	-
StyGPx8	Contig19413.g20586	21.3	0	-	-
StyGPx9	Contig19790.g20991	20.5	1	-	-
EvGPx1	MSTRG.6189	16.8	1	?	-
EvGPx2	MSTRG.7263	22.1	0	?	-
SteGPx1	SteCoe_16643	19.8	?	-	+
SteGPx2	SteCoe_21264	18.4	?	-	+
SteGPx3	SteCoe_26857	21.5	0	-	+
SteGPx4	SteCoe_40903	18.7	?	-	+
SteGPx5	SteCoe_40904	19.0	?	-	+
SteGPx6	SteCoe_40905	20.0	?	-	+
SteGPx7	SteCoe_6726	20.2	?	-	+
McGPx1	ACL81236.1	21.3	0	-	+
McGPx2	AFR60589.1	21.2	0	-	+
McGPx3	ACL81237.1	21.2	0	-	+
PcpGPx1	PPERSA_00007440	17.1	2	?	-
PcpGPx2	PPERSA_00008250	25.7	3	?	-
PcpGPx3	PPERSA_00077430	13.3	2	?	-
PcpGPx4	PPERSA_00077470	20.2	4	?	-
PcpGPx5	PPERSA_00073590	26.2	4	?	-
PcpGPx6	PPERSA_00077460	14.5	3	?	-
PcpGPx7	PPERSA_00040530	27.3	2	?	-
EocGPx1	Contig11975.g1890	21.4	0	+	-
EocGPx2	Contig13463.g3154	21.1	0	-	-
EocGPx3	Contig23176.g11961	45.1	4	-	-
EocGPx4	Contig32168.g20842	21.1	0	-	-
EocGPx5	Contig8754.g27852	22.7	1	-	-
**Other selected organisms**					
CrGPx5	AAB66330.1	18.0	4	-	-
CrGPx1	AAL14348.1	21.4	0	+	+
TcGPx1	TcCL_NomESM05066	17.4	0	?	-
PfGPx1	PF3D7_1212000	23.9	2	?	-
ScGPx1	EDV09454.1	18.6	0	-	-
AtGPx6	AEE83029.1	25.5	0	-	-
DmGPx1	NP_728869.1	26.2	0	-	-
HsGPx1	NP_000572.2	22.0	0	+	+
HsGPx5	NP_001500.1	25.2	0	-	-
HsGPx4	AAH32695.3	22.1	0	+	+

^(1)^ Accession numbers were obtained from different databases (see bioinformatics analysis section in the manuscript). MW: molecular weight. Sec: selenocysteine. (+): presence. (-): absence. (?): It has not been possible to analyze, because the UTR sequences are not available. ^(2)^ There are two versions of this gene, one with only one intron and the other with two introns. Tt: *T. thermophila*. Tb: *T. borealis*. Te: *T. ellioti*. Tm: *T. malaccensis*. Im: *Ichthyophthirius multifiliis*. Pt: *Paramecium tetraurelia*. Oxy: *Oxytricha trifallax*. Sty: *Stylonychia lemnae*. Ev: *Euplotes vannus*. Ste: *Stentor coeruleus*. Mc: *Monoeuplotes crassus*. Pcp: *Pseudocohnilembus persalinus*. Eoc: *Euplotes octocarinatus*. Cr: *Chlamydomonas reinhardtii*. Tc: *Trypanosoma cruzi*. Pf: *Plasmodium falciparum*. Sc: *Saccharomyces cerevisiae*. At: *Arabidopsis thaliana*. Dm: *Drosophila melanogaster*. Hs: *Homo sapiens*. Positives are highlighted in green. The GPx from the different species are highlighted in white or blue.

**Table 2:** Catalytic tetrad conserved motifs from putative ciliate GPx.

Catalytic Tetrad ^(1)^	Ciliate GPx
CXC/Q/W/N	TtGPx1, 2, 4, 5, 6, 7, 8, 9
	TbGPx1, 2, 3, 4
	TeGPx1, 2, 3, 4, 5, 6
	TmGPx1, 2, 3, 4, 5, 6, 7
XXC/Q/W/N	OxyGPx6
**C** **S** **C/Q/W/N**	PtGPx2, 3, 4, 5
SXC/Q/W/N	TtGPx3 TbGPx5 TeGPx7 TmGPx8 StyGPx2, 3, 4 OxyGPx3, 5, 8 EvGPx2 EocGPx1, 2, 4
CXU/Q/W/N	TtGPx12
SXU/Q/W/N	SteGPx1, 4, 5, 7 McGPx1, 2, 3
SXU/X/W/N	SteGPx2
CXU/X/X/N	SteGPx3, 6
SXX/Q/W/N	TmGPx10 StyGPx9 OxyGPx7
SXS/X/X/N	OxyGPx4
XXX/Q/X/N	EvGPx1
XXX/Q/W/N	TbGPx7 TeGPx8, 9 TmGPx9 ImGPx1 StyGPx1, 6, 7 OxyGPx9 PcpGPx4, 6
CXX/Q/W/N	TbGPx6
SXC/X/X/X	PcpGPx3
SXC/X/W/N	PcpGPx1
SXX/Q/X/N	StyGPx8
XXX/X/W/N	OxyGPx2

^(1)^ Although the GPx catalytic tetrad consists of four amino acids (C/U, Q, W and N), highlighted in red, we also consider the two contiguous residues to the left of the first tetrad’s amino acid: Serine (S) highlighted in green and cysteine (C) in blue. (C): cysteine, (U): selenocysteine, (Q): glutamine, (W): tryptophan, (N): asparagine, (S): serine, (X): any other amino acid. See Table 1 for species names.

**Table 3:** Ranking of gene expression induction values.

Treatments	TtGPx Genes
H_2_O_2_ (1 h)	*TtGPx1 >> TtGPx3 > TtGPx9 > TtGPx10 > TtGPx12 > TtGPx11*
MD (1 h)	*TtGPx9 >> TtGPx1 > TtGPx3 > TtGPx10 > TtGPx11 > TtGPx12*
PQ (1 h)	*TtGPx9 >> TtGPx1 > TtGPx10 > TtGPx12 > TtGPx11 > TtGPx3*
PQ (24 h)	*TtGPx1 > TtGPx9 > TtGPx12 > TtGPx10 > TtGPx3 > TtGPx11*
CAM (1 h)	*TtGPx12 > TtGPx3 > TtGPx10 > TtGPx9 > TtGPx11 > TtGPx1*
CAM (24 h)	*TtGPx9 > TtGPx3 > TtGPx10 > TtGPx12 > TtGPx11 > TtGPx1*
Cd (1 h)	*TtGPx9 >> TtGPx1 > TtGPx10 > TtGPx3 > TtGPx12 > TtGPx11*
Cd (24 h)	*TtGPx12 >> TtGPx9 > TtGPx10 > TtGPx3 > TtGPx1 > TtGPx11*
Pb (1 h)	*TtGPx1 > TtGPx9 > TtGPx3 > TtGPx12 > TtGPx10 > TtGPx11*
Pb (24 h)	*TtGPx12 > TtGPx9 > TtGPx10 > TtGPx1 > TtGPx3 > TtGPx11*
Cu (1 h)	*TtGPx12 > TtGPx3 > TtGPx10 > TtGPx9 > TtGPx11 > TtGPx1*
Cu (24 h)	*TtGPx10 > TtGPx12 > TtGPx3 > TtGPx9 > TtGPx11 > TtGPx1*

MD: menadione. PQ: paraquat. CAM: camptothecin. Each type of analyzed TtGPx is identified by different colors.

## Supplementary Materials

The following are available online at https://www.mdpi.com/2076-2607/8/7/1008/s1, Figure S1: Multiple-sequence alignment of 101 GPx from ciliates and other organisms. Only regions containing residues of the catalytic tetrad (U/C, Q, W, N) are shown, indicated by ▼. Highly conserved residues in the regions adjacent to the tetrad are indicated by gray shading and +. See Table 1 for species names. Figure S2: Phylogram of all *T. thermophila* GPx amino acid sequences. Catalytic tetrads are indicated. Numbers indicate bootstrap values from 2000 replicates. Branch lengths are drawn to scale, as indicated by the scale bar. Figure S3: Phylogram of all ciliate putative GPx isoforms. Catalytic tetrads are indicated. No residues in the GPx catalytic tetrad are highlighted within a red square. Species with SECIS elements are indicated by a red star. Numbers indicate bootstrap values from 2000 replicates. Branch lengths are drawn to scale, as indicated by the scale bar. See Table 1 for ciliate species identification. Table S1: Primers used in the qRT-PCR analysis. Table S2: Quantitative RT-PCR standard curve parameters. Table S3: Characteristics of ciliate GPx SECIS elements. Table S4: Secondary structure characteristics of ciliate SECIS elements. Table S5: Relative fold induction values ± SD of selected TtGPx genes, under different stressful conditions.
