# Supplementary material for: Tetrahymena Glutathione Peroxidase Family: A Comparative Analysis of These Antioxidant Enzymes and Differential Gene Expression to Metals and Oxidizing Agents
Source: Microorganisms. 2020 Jul 5;8(7):1008. doi: 10.3390/microorganisms8071008 (PMC7409322; doi:10.3390/microorganisms8071008)
Supplement: Supplementary file 1 [file microorganisms-08-01008-s001.pdf]

Table S1. Primers used in the qRT-PCR analysis

Primers	Sequence (5´- 3´)	Amplified gene
αTub1	TGTCGTCCCCAAGGAT	α-tubulin
αTub2	GTTCTCTTGGTCTTGATGGT	
βact1	CTCTCTTTCTACCTTCCAAACT	β-actin
βact2	AGGACCAGATTCATCATATTC	
GPx1A	CTTGGGCTGAGTCTGAAA	*TtGPx1*
GPx1B	GAAAAGAGGGAATTCTACGT	
GPx3A	TAGCAAATAATACTCCTGGAT	*TtGPx3*
GPx3B	GGTTTTCCCTCAGCATT	
GPx9A	CCCGATGGTAAGGTTCA	*TtGPx9*
GPx9B	CAGCTATCATATCGTTAGGAT	
GPx10A	GTGATGGTACAGTTCATAGTT	*TtGPx10*
GPx10B	AGGTTCAATTTCTTTTGGTT	
GPx11A	AAGGTAGAGGTAAATGGTTCA	*TtGPx11*
GPx11B	AGAAGAATGTCTCTTCAAGAA	
GPx12A	CTAGTATACAAGATGATTCTG	*TtGPx12*
GPx12B	GTCTCTACTGAAAAGCTTAAT	

Table S2. Quantitative RT-PCR standard-curve parameters

Gene	Slope	Efficiency (%)	R^2 [(1)]
α-tubulin	- 3.43	95.56	0.99
β-actin	- 3.35	98.98	0.99
TtGPx1	- 3.47	93.96	0.99
TtGPx3	- 3.58	90.16	0.99
TtGPx9	- 3.52	92.24	0.99
TtGPx10	- 3.50	92.92	0.99
TtGPx11	- 3.42	96.14	0.99
TtGPx12	- 3.33	99.77	0.99

[(1)] Correlation coefficient. Efficiency (E) is calculated from the slope value of the standard curve: $E = 10^{(-1/slope)-1}$

Table S3. Characteristics of ciliate GPx SECIS elements.

GPx	Location(1)	Length (b)	DNA strand(2)	Type(3)	Score(4)	Grade(5)
TtGPx10	3´UTR (96 b -> Stop)	82	+	I	22.19 (C)	A
TbGPx6	3´UTR (63 b -> Stop)	77	+	I	14.89 (I)<br>27.17 (C)	A
TbGPx7	3´UTR (121 b -> Stop)	74	+	I	13.97 (I)<br>21.84 (C)	A
TmGPx9	3´UTR (48 b -> Stop)	74	+	I	13.54 (I)<br>23.89 (C)	A
TmGPx10	3´UTR (99 b -> Stop)	82	+	I	30.28 (C)	A
EocGPx1	3´UTR (29 b -> Stop)	71	+	I	14.49 (C)	A
TeGPx8	2º intron	75	+	I	19.34 (I)<br>29.61 (C)	A
StyGPx5	ORF (337 b -> ATG)	59	-	I	13.89 (I)<br>1.35 (C)	B
StyGPx6	5´UTR (105 b -> Tel)	71	-	I	18.71 (I)<br>14.89 (C)	B

(1) Nine possible SECIS locations have been detected: in the 3'UTR region (the number of bases (b) from the stop codon to the SECIS element is indicated), within an intron (the intron number is indicated), in the ORF or coding region (the number of bases from the start codon to the SECIS element is indicated), or in the 5'UTR region (the number of bases from the telomere to the SECIS element is indicated). (2) Strand on which the SECIS element was found: (+): on the sequence as it was input, (-): on its reverse complement. (3) Eukaryotic SECIS Type-I or II. (4) The search method(s) predicted the SECIS element: by Covels (C) program and/or Infernal (I) program. (5) Marker for how good the SECIS prediction looks like. The SECIS grade can be A, B, C in decreasing order of goodness.

Table S4. Secondary structure characteristics of ciliate SECIS elements.

GPx	SECIS core [(1)] (UGAN/KGAW)	Conserved A in Loop-II	Stem-I (bp)	Stem-II [(2)] (bp)	Loop-I (b)	Loop-II (b)	Mismatches bases (bp)	Non-canonical pairing (AG/GA)
TtGPx10	UGAA/UGAA	3	7	11	21	13	2	0
TbGPx6	UGAC/UGAA	3	6	11	11	16	1	0
TbGPx7	UGAC/UGAA	2	6	11	12	14	2	0
TmGPx9	UGAU/UGAA	3	11	11	6	15	2 + 1b	1
TmGPx10	UGAA/UGAA	3	7	11	19	15	2	0
EocGPx1	UGAU/UGAA	3	8	10	10	10	1 b	1
TeGPx8	UGAU/UGAA	3	11	12	6	15	1	1
StyGPx5	UGAU/UGAG	0	8	8	13	2	1	1
StyGPx6	UGAC/AGAU	3	9	11	6	13	2	1

(1) K: (U/G), W: (U/A), N: any nucleotide. (2) After removing the SECIS core.

Table S5. Relative-fold induction values ± SD of selected *TtGPx* genes, under different stressful conditions.

Treatment	TtGPx gene					
	TtGPx1	*TtGPx3*	*TtGPx9*	*TtGPx10*	*TtGPx11*	*TtGPx12*
H_2O_2 (1h)	74.76 ± 1.45	9.09 ± 0.15	6.26 ± 0.89	5.77 ± 0.09	1.94 ± 0.06	4.9 ± 0.88
MD (1h)	733.34 ± 67.4	45.59 ± 2.91	3802 ± 296.17	40.62 ± 5.23	6.91 ± 0.55	3.24 ± 1.78
PQ (1h)	224.9 ± 6.93	7.42 ± 0.22	664.95 ± 16.9	52.14 ± 2.43	8.21 ± 0.28	26.21 ± 0.95
PQ (24h)	218.58 ± 80.31	33.19 ± 3.46	168.46 ± 58.79	58.34 ± 21.01	29.9 ± 3.72	113.92 ± 11.86
CAM (1h)	0.91 ± 0.09	9.22 ± 1.38	6.31 ± 0.63	9.17 ± 2.3	4.14 ± 0.58	13.71 ± 2.1
CAM (24h)	19.62 ± 0.72	561.87 ± 24.65	1996.27 ± 93.69	312.16 ± 84.8	25.11 ± 1.07	237.42 ± 15.36
Cd (1h)	949.11 ± 59.1	156.13 ± 26.67	2175.83 ± 656.27	216.16 ± 63.98	26.97 ± 8.34	81.01 ± 41.45
Cd (24h)	15.22 ± 1.05	17.87 ± 0.98	42.22 ± 2.57	29.64 ± 6.28	9.78 ± 0.75	318.12 ± 17.12
Pb (1h)	141.44 ± 3.94	73.04 ± 2.2	138.27 ± 5.65	41.79 ± 1.08	18.74 ± 0.56	45.25 ± 6.32
Pb (24h)	119.55 ± 7.25	52.06 ± 1.85	134.88 ± 7.07	126.84 ± 6.39	24.2 ± 1.2	158.88 ± 8.87
Cu (1h)	2.98 ± 0.61	47.67 ± 8.76	12.39 ± 2.22	36.98 ± 7.24	10.4 ± 1.97	71.2 ± 7.22
Cu (24h)	2.67 ± 0.42	33.85 ± 4.01	25.83 ± 3.39	64.79 ± 6.87	9.97 ± 2.14	35.9 ± 3.03

SD: Standard deviation. MD: menadione. PQ: paraquat. CAM: camptothecin.

Figure S1. Multiple-sequence alignment of 101 GPx from ciliates and other organisms. Only regions containing residues of the catalytic tetrad (U/C, Q, W, N) are shown, indicated by ▼. Highly conserved residues in the regions adjacent to the tetrad are indicated by grey shading and +. See Table 1 for species names.

```
             | ....|....| ....|....|
            940       950       960
                     +++  ▼
TtGPx1      K KAIIVVNVAC QCGLTSD---
TtGPx2      K KAIIVVNVAC KCGLTSG---
TtGPx3      K KAYLIVNVAS KCGFTST---
TtGPx4      K KAIIVVNVAC KCGLTSD---
TtGPx5      K KVIIVVNVAC KCGLTSD---
TtGPx6      K KAIIVVNVAC KCGLTSD---
TtGPx7      K KCILVVNVAC KCGLTSD---
TtGPx8      K KCILVVNVAC KCGLTSD---
TtGPx9      R KCLLVVNVAC KCGLTSD---
TtGPx10     Q KLLTLMEIYV KCQSFKI---
TtGPx11     N KYAIVVNTGS QNPNFKQ---
TtGPx12     K KAIIIVNVAC KUGLTGD---
TbGPx1      K KAIIVVNVAC KCGLTSD---
TbGPx2      K KVILVVNVAC KCGLTSA---
TbGPx3      K KCILVVNVAC KCGLTSE---
TbGPx4      K KCILVVNVAC KCGLTSD---
TbGPx5      K KAYLIVNVAS KCGFTST---
TbGPx6      K KAIIIVNVAC ----------
TbGPx7      R KAVIVVNVAL ----------
TbGPx8      N KYAIIVNTGS QNPKFKQ---
TeGPx1      K KAIIVVNVAC KCGLTSD---
TeGPx2      K KAIIVVNVAC QCGLTSD---
TeGPx3      R KCLLVVNVAC KCGLTSD---
TeGPx4      K KCILVVNVAC KCGLTSD---
TeGPx5      K KCILVVNVAC KCGLTSD---
TeGPx6      K KCILVVNVAC KCGLTSD---
TeGPx7      K KAYLIVNVAS KCGFTST---
TeGPx8      K KAIIIVNVAY ----------
TeGPx9      Q KKIYVFNLNL ----------
TeGPx10     N KYAIVVNTGS QNPNFKQ---
TmGPx1      K KVIIVVNVAC KCGLTSD---
TmGPx2      K KAIIVVNVAC KCGLTSD---
TmGPx3      K KAIVVVNVAC KCGLTSD---
TmGPx4      R KCLLVVNVAC KCGLTSD---
TmGPx5      K KCILVVNVAC KCGLTSD---
TmGPx6      K KCILVVNVAC KCGLTSD---
TmGPx7      K KCILVVNVAC KCGLTSD---
TmGPx8      K KAYLIVNVAS KCGFTST---
TmGPx9      - ---------- ----------
TmGPx10     R KALIVVNVAL ----------
TmGPx11     N KYAIVVNTGS QNPNFKQ---
ImGPx1      - ---------- ----------
ImGPx2      N KVVIVVNLGS QNNCYQE---
StyGPx1     - --------MV ----------
StyGPx2     P KLSIVVNVAS ECGLTES---
StyGPx3     K KCTIIVNVAS KCGLSQK---
StyGPx4     P KLSLVVNVAS RCGLTDS---
StyGPx5     - KVILFTNLSI EESQKEEITE
StyGPx6     K RCTLVS---- ----------
StyGPx7     - --------LV ----------
StyGPx8     K KCILVTNTAS LSPHANM---
StyGPx9     K RCILVTNVAS ----------
```

```
             | ....|....| ....|....|
            940       950       960
                     +++  ▼
OxyGPx1     - KVLLFVNMSV EDKQKLEESE
OxyGPx2     - ---------- ----------
OxyGPx3     P KATLIANVAS NCGFTNN---
OxyGPx4     K KCTMVVNVAS QSKFAAT---
OxyGPx5     P QVTMIVNVAS KCGHTKK---
OxyGPx6     P KLAIVVNVAT KCGLTKG---
OxyGPx7     K SCILVVNVAS ----------
OxyGPx8     P KLSLFVNVAS KCGLTDS---
OxyGPx9     K RCILVT---- ----------
EvGPx1      - ---------- ----------
EvGPx2      Y KAILIVNVAS KCGLTKG---
SteGPx1     - KVLIIVNVAS AUARTAR---
SteGPx2     - KALLIVNIAS EURKTSS---
SteGPx3     - KILLIVNIAC KUHMSGK---
SteGPx4     - KVLLIVNVAS EUGLTKK---
SteGPx5     - HILLIVNVAS QUELTKI---
SteGPx6     - KVLVIVNIAC KUQLSPK---
SteGPx7     - KVLIIVNVAS QUGKTRR---
PtGPx1      Q KVVI-VNVAI DSPELND---
PtGPx2      K KAYICVNVAC SCGLTSS---
PtGPx3      K KVIICVNVAC SCGLTSG---
PtGPx4      K KAYICVNVAC SCRLTTQ---
PtGPx5      K KAYICVNVAC SCGLTSS---
McGPx1      K KCIMVVNVAS KUGLTKT---
McGPx2      R KCTMVVNVAS KUGLTKT---
McGPx3      R KCTMVVNVAS KUGLTKT---
PcpGPx1     Y KIIMIVNVAS ACGTID----
PcpGPx2     S WGILFSHPDA FTPICT---S
PcpGPx3     K DVYLLVNVAS ECD-------
PcpGPx4     - ---------- --SLTVK---
PcpGPx5     S WAILFSHPAD FTPVCT---T
PcpGPx6     - ---------- ----------
PcpGPx7     K -IILCTNVSS RGSMAKE---
EocGPx1     C KCIMVVNVAS KCSLTNK---
EocGPx2     C KCVMVVNVAS ECGLTKT---
EocGPx3     - ---------- ----------
EocGPx4     Y KCIMVVNVAS GCGYTDT---
EocGPx5     - QVTLYSNICH NDKESKA---
CrGPx5      - KAVLIVNVAS KCGFTP----
CrGPx1      - RVVLVVNVAS KUGLTAA---
ScGPx1      - KVVLIVNVAS KCGFTP----
TcGPx1      - RVTVVVNTAS LCSFANS---
PfGPx1      - KVLIIFNSAS KCGLTKN---
AtGPx6      - KVLLIVNVAS QCGLTNS---
DmGPx1      - KVVLVVNIAS KCGLTKN---
HsGPx1      - KVLLIENVAS LUGTTVR---
HsGPx5      - KHILFVNVAT YCGLT-A---
HsGPx4      - FVCIVTNVAS QUGKTEVNYT
```

```
             ....|....| ....|....| ....|....|
                  1030       1040
                  ++ ++  +++   ▼++      +
TtGPx1       SQGLEILGFP CNQFGAQEPW AESEILSYTQ
TtGPx2       SQGLEVLAFP CNQFGEQEPW AESEILSYTQ
TtGPx3       DKGLEILAFP SNQFFNQEPF DEPAIKEFVK
TtGPx4       SQGLEILAFP CNQFGQQEPW AESEILSYTQ
TtGPx5       SQGLEVLAFP CNQFGEQEPW AESEILSYTQ
TtGPx6       SQGLEVLAFP CNQFGEQEPW AESEILSYTQ
TtGPx7       SRGFEILAFP TNDFMEQEPW DNKKIKEYVQ
TtGPx8       SRGFEILAFP TNDFMEQEPW DNNKIKEYVQ
TtGPx9       SQGFEILAFP ANQFMGQEPW DNAKIKEYVV
TtGPx10      HLTTTLSQWK CIRNTNREVW KFLHSLPINL
TtGPx11      KDKLEILAFP CNQFYN-EPS NFKTIKDSYS
TtGPx12      DSGLEILGFP CNQFMSQEPW AEPKIKDFIT
TbGPx1       AQGLEILGFP CNQFGAQEPW DEKEILSYTK
TbGPx2       DQGLEILGFP CNQFLSQEPW DEPKIQEFIK
TbGPx3       TQGFEILAFP ANQFMGQEPW DNAKIKEYIV
TbGPx4       NRGFEILAFP TNDFMQQEPW DSKKIKEYVQ
TbGPx5       DQGLEILAFP SNQFMNQEPF DEPQIKEFVK
TbGPx6       ---LEILGFP CNQFLSQEPW AEPKIKDFIT
TbGPx7       SRGLEILAFP SNQFMGQEPW DPPQIKEFVV
TbGPx8       KDQLEVIAFP CNQFYN-EPS NFKGIQENYA
TeGPx1       QQGLEILGFP CNQFGAQEPW SESEILSYTQ
TeGPx2       SQGLEILGFP CNQFGSQEPW AESEILSYTQ
TeGPx3       SQGFEVLAFP ANQFMGQEPW DNAKIKEYTV
TeGPx4       SRGFEILAFP TNDFMEQEPW DNQKIKEYVQ
TeGPx5       SKGFEILAFP TNDFMQQEPW DNKKIKEYVQ
TeGPx6       SKGFEILAFP TNDFMEQEPW DNKKIKEYVQ
TeGPx7       DKGLEILAFP SNQFLNQEPF DEPQIKEFVK
TeGPx8       -HGLEVLGFP CNQFMSQEPW AEPKIKEFIT
TeGPx9       SRGLEILAFP SNQFMEQEPW DPPQIKEFVV
TeGPx10      NDKLEILAFP CNQFYN-EPS NFKTIKEQYS
TmGPx1       SQGLEILGFP CNQFGAQEPW SESEILSYTQ
TmGPx2       SQGLEILAFP CNQFGQQEPW AESEILSYTQ
TmGPx3       TQGLEILAFP CNQFGQQEPW DESEILSYTQ
TmGPx4       SQGFEILAFP ANQFMGQEPW DNAKIKEYVV
TmGPx5       SRGFEILAFP TNDFMEQEPW DNKKIKEYVQ
TmGPx6       SRGFEILAFP TNDFMEQEPW DNKKIKEYVQ
TmGPx7       SRGFEILAFP TNDFMEQEPW DNKKIKEYVQ
TmGPx8       DKGLEILAFP SNQFLNQEPF DEPAIKEFVK
TmGPx9       DSGLEILGFP CNQFMSQEPW AEPKIKDFII
TmGPx10      SRGLEILAFP SNQFMGQEPW DPPQIKEFVV
TmGPx11      KDKLEILAFP CNQFYN-EPL NFKNIKDSYS
ImGPx1       ---FTILAFP ANQFMSQEPW DPPQIKDFVI
ImGPx2       -----------ANQ------- ----------
StyGPx1      EQGFEILAFP VNQFFSQEPG TNQQIKSLVR
StyGPx2      DQGFEVLAFP CNQFGAQEPG SNSQIFEFVC
StyGPx3      DQGLEILAFP CNQFMNQEPG SNLQILEYAR
StyGPx4      PQGFEVMAFP CNQFGSQEPG TNKQILDFVC
StyGPx5      ERGFCVYGAL PNDVAGGNPL RNKEIANTLK
StyGPx6      DQGFEILTFP CNQFGGQEPG TNQEVKQFIR

             ....|....| ....|....| ....|....|
                  1030       1040
                  ++ ++  +++   ▼++      +
StyGPx7      EKGFEILAFP CNQFMKQEPK TNQEIKTFLQ
StyGPx8      SKGLEILAFP CNQFHKQEPL NDNEIKVNAQ
StyGPx9      DQGFEILAFP CNQFMSQEPG SNQEIKQFVR
OxyGPx1      ---------- ----ESGNPL RNRDLAILLK
OxyGPx2      DQGFEILAFP SNEFFSTEPG DSKQIKSLVR
OxyGPx3      QHGLEIVGFP CNQFFSQEKG TNDEIFQFVC
OxyGPx4      AQGFEILAFP CNQFNKEEPW NDHEIKQHVI
OxyGPx5      DQGFEIVAFP CNQFGSQESK PNETIYEFVC
OxyGPx6      SQGFDVLAFP CNQFLFQEPG SAEEIQKFAC
OxyGPx7      DKGFEILAFP CNQFMRQEPR SNQEIKEYVI
OxyGPx8      EQGFEVLAFP CNQFGSQEPD SNASILDFVC
OxyGPx9      DQGFEILAFP CNQFMNQEPG TPEEIKKFIK
EvGPx1       HKGFQILAFP CNQFLGQESC SNDDINEFVR
EvGPx2       DKGFQIFAFP CNQFGAQEPG TNEEIRAFAQ
SteGPx1      SEGLCILAFP CNQFFHQEAG TSSEIKDYIR
SteGPx2      DKEFEILGFP CNQFNNREPG TDEQIVAFAK
SteGPx3      SRGLEILSFP CNQFFWREYS EQETIKSYLI
SteGPx4      SKGLEILAFP CNQFLHQEPG SDEEIEKFAR
SteGPx5      KEGLIIALFP CNQFNNQEPG TNAEIQEFIR
SteGPx6      SRGLEILAFP CSQFFWREYS THNEIKDYLS
SteGPx7      SRGLCILAFP CNQFFYQERG TSDQIKDFIT
PtGPx1       -LPYQVLLFP KCDH----TF TY------KQ
PtGPx2       AQGLEILGFP CNQFMGQESK PEPEIKEFVL
PtGPx3       AQGLEILGFP CNQFMNQESK PEPEIKEFVI
PtGPx4       DQGLEILGFP CNQFRNQESK PEPEIKNYVT
PtGPx5       AQGLEILGFP CNQFMGQESQ PEPEIKEFVI
McGPx1       DKGFEIFAFP CNQFLSQEPG SNEDIKKFAR
McGPx2       DQGFEIFAFP CNQFMSQEPG THEQIKKFAQ
McGPx3       DHGFEIFAFP CNQFMSQEPG THEQIKKFAQ
PcpGPx1      ---------- ---------- ---EIKQFLN
PcpGPx2      KREVKVVAIS CNDVKTHKDW IKD-IEHY--
PcpGPx3      ---------- ---------- ---EIQHVVR
PcpGPx4      DQGFEILAFP CNQFGKQEPL PNDQILEAAR
PcpGPx5      KKGVKVIALS CNDADTHKEW IKD-VDHY--
PcpGPx6      QKGLEIFAFP CNQFGAQEPN PNNEILDAAR
PcpGPx7      NNGFEILCFP SNQFYN-EPG TFSNLKQIYL
EocGPx1      DKGFEIFAFP CNQFLSQESC SNEHIKNFVQ
EocGPx2      DKGFQIFAFP CNQFMKQEPG SNEDIKKFAQ
EocGPx3      ---------- ---------- ----------
EocGPx4      DKGFQIFAFP CNQFMGQESK CNLDIKKYAQ
EocGPx5      DKGLRVFGLP TTDVLIGSTK LTYD------
CrGPx5       DRGLVILGFP CNQFGGQEPG DASAIGEFCQ
CrGPx1       ATDLTIVAFP CNQFGGQEPG TNAEIKAFAS
ScGPx1       DEGFTIIGFP CNQFGHQEPG SDEEIAQFCQ
TcGPx1       PRGFTILAFP CAQFANQEPK SNEEIAVWAQ
PfGPx1       ARGLEILAFP TSQFLNQEFD NTKDICTFNE
AtGPx6       GHGFEILAFP CNQFGNQEPG TNEEIVQFAC
DmGPx1       ERGLVILNFP CNQFGSQMPE ADGEAMVCHL
HsGPx1       PRGLVVLGFP CNQFGHQENA KNEEILNSLK
HsGPx5       PYGLVVLGFP CNQFGKQEPG DNKEILPGLK
HsGPx4       EFAAGYNVKF DMFSKICVNG DDAHPLWKWM
```

```
              .|....| ....|....|
                  1140      1150
                 + ▼ ▼+  +++
TtGPx1        AATKIPW NF-AKFLVDG
TtGPx2        SATKIPW NF-AKFLIDG
TtGPx3        FQGYIQW NF-AKFLVNA
TtGPx4        SATKIPW NF-AKFLIDG
TtGPx5        YATKIPW NF-AKFLIDG
TtGPx6        SATKIPW NF-AKFLIDG
TtGPx7        KTRQIPW NF-AKFLIDP
TtGPx8        KTRQIPW NF-AKFLINP
TtGPx9        KTRQIPW NF-AKFLIGP
TtGPx10       NLAQILF SL-VKQMLTE
TtGPx11       NGAKITE DF-SKFLINT
TtGPx12       KAKQIPW NF-SKFVVDR
TbGPx1        AAQKIPW NF-AKFLIDG
TbGPx2        KSKQIPW NF-GKFIINK
TbGPx3        KTRQIPW NF-AKFLIGP
TbGPx4        KTRQIPW NF-AKFLIDP
TbGPx5        FQGYIQW NF-AKFLINS
TbGPx6        KAKQIPW NF-SKFLVNQ
TbGPx7        KAKQIPW NF-AKFLIQP
TbGPx8        NGEKITE DF-TKFLVNT
TeGPx1        AATKIPW NF-AKFLVDG
TeGPx2        AATKIPW NF-AKFLIDG
TeGPx3        KTRQIPW NF-AKFLIGP
TeGPx4        KTRQIPW NF-AKFLINP
TeGPx5        KTRQIPW NF-AKFLINP
TeGPx6        KTRQIPW NF-AKFLINP
TeGPx7        FQGFIQW NF-AKFLVDG
TeGPx8        KAKQIPW NF-SKFIINR
TeGPx9        NSKQIPW NF-AKFLIQN
TeGPx10       GGIKITE DF-SKFLINN
TmGPx1        AATKIPW NF-AKFLIDG
TmGPx2        SATKIPW NF-AKFLIDG
TmGPx3        SATKIPW NF-AKFLIDG
TmGPx4        KTRQIPW NF-AKFLIGP
TmGPx5        KTRQIPW NF-AKFLIDP
TmGPx6        KTRQIPW NF-AKFLINP
TmGPx7        KTRQIPW NF-AKFLIDP
TmGPx8        FQGYIQW NF-AKFLVNA
TmGPx9        KAKQIPW NF-SKFVVDR
TmGPx10       NSKQIPW NF-AKFLIQS
TmGPx11       NGAKITE DF-SKFLINT
ImGPx1        KAKNIPW NF-GKFLINK
ImGPx2        NGIKINE DF-CKFLVDQ
StyGPx1       QVKRIPW NF-SKFLVNK
StyGPx2       GLDQVTW NF-GKFLIDQ
StyGPx3       KVKALPW NF-CKFILDQ
StyGPx4       RLARITW NF-GKFLVNQ
StyGPx5       KAIRVKE NY-SKLLCNR
StyGPx6       EVKEIPW NF-AKFLVNE
StyGPx7       DIRMIPW NF-SKFLVDS
StyGPx8       KIKDIPG NF-TKFLVDR
StyGPx9       EVKEIPW NF-AKFLVNE
```

```
              .|....| ....|....|
                  1140      1150
                 + ▼ ▼+  +++
OxyGPx1       RAYRIKE SY-AKFLCNR
OxyGPx2       KVKQIPW NF-SKFLVDS
OxyGPx3       NFNKIGW NF-GKFLVNQ
OxyGPx4       NIKEIPG NF-AKFLVDR
OxyGPx5       KVGKIPW NF-AKFLGDQ
OxyGPx6       GITRIEW NF-GKFLVDS
OxyGPx7       GVKEVPW NF-SKFLVNK
OxyGPx8       RIAKITW NF-GKFLVNK
OxyGPx9       EVKEIPW NF-AKFLVDQ
EvGPx1        KLKSIPF NF-SKFLINS
EvGPx2        TTGDIPW NF-AKFLINS
SteGPx1       DGKRICW NF-GKFLVDR
SteGPx2       KGSGIDW NF-GKFLVDK
SteGPx3       NGRKIGM NF-CKFLVDR
SteGPx4       DGGKIGW NF-GKFIVSR
SteGPx5       KGREIGW NF-TKFLVNR
SteGPx6       NSTQIGL NF-GKFIVDK
SteGPx7       NRRKICW NF-GKFLIDR
PtGPx1        NGRQIKQ DF-CKFLISE
PtGPx2        EAKEVPW NF-GKFLLNS
PtGPx3        SARQVPW NF-GKFLLDS
PtGPx4        EVKYVPW NF-AKFLLDA
PtGPx5        EAKEVPW NF-GKFLLNS
McGPx1        TIQNIPW NF-AKFLIDE
McGPx2        VVQNIPW NF-AKFLIDN
McGPx3        VVQNIPW NF-AKFLIDE
PcpGPx1       NTNNIPW NF-AKFVVYD
PcpGPx2       DPQGLPM TVRSVYVIGP
PcpGPx3       ENPNIAY ----VTIKSN
PcpGPx4       KTKNILW NF-GKFLIDK
PcpGPx5       DAKGLPM TVRSVYIIGP
PcpGPx6       KCEDIKW NF-GKFLVDG
PcpGPx7       SAEPIVE DF-SKFLIDQ
EocGPx1       TMQSIPW NF-TKFLIDE
EocGPx2       TLQNIPW NF-AKFLIDE
EocGPx3       ---------- -------
EocGPx4       EIQSIPW NF-TKFLIND
EocGPx5       KSRRINT HF-NKFLCDR
CrGPx5        MMEMIKW NF-EKFLVDK
CrGPx1        --SDIGW NF-GKFLVRP
ScGPx1        GLRGIKW NF-EKFLVDK
TcGPx1        --GPIRW NY-TKFICDR
PfGPx1        TLKSIGW NF-GKFLVDK
AtGPx6        -GDGIKW NF-AKFLVDK
DmGPx1        -GSGIKW NF-TKFLVNK
HsGPx1        CRNDVAW NF-EKFLVGP
HsGPx5        KVHDIRW NF-EKFLVGP
HsGPx4        LGNAIKW NF-TKFLIDK
```

Figure S2. Phylogram of all *T. thermophila* GPx amino acid sequences. Catalytic tetrads are indicated. Numbers indicate bootstrap values from 2000 replicates. Brach lengths are drawn to scale as indicated by the scale bar.

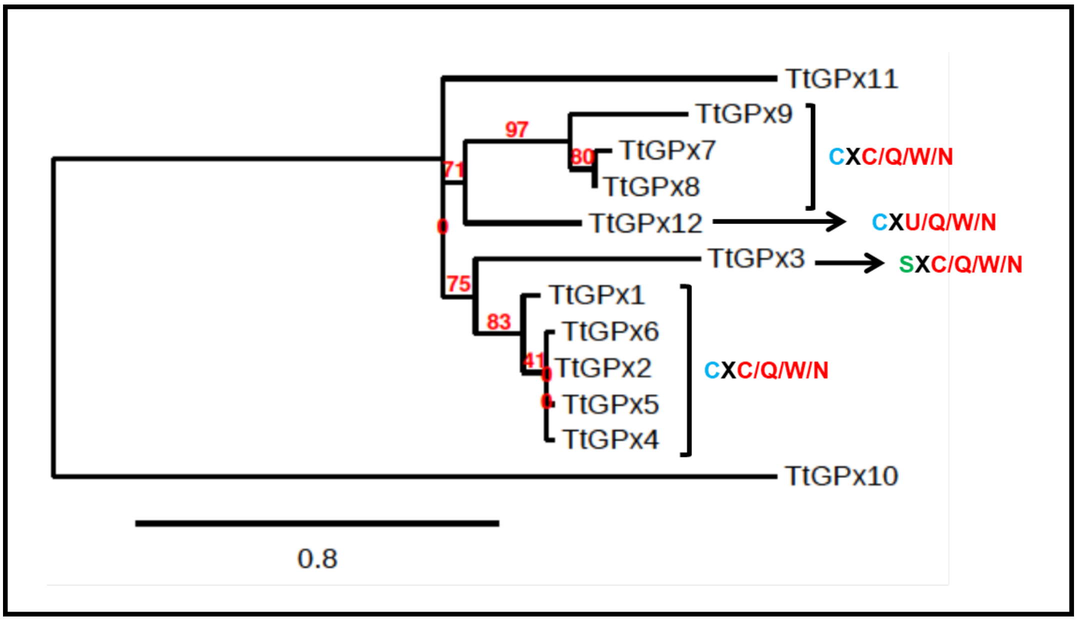

Figure S3. Phylogram of all ciliate putative GPx isoforms. Catalytic tetrads are indicated. No residue of the GPx catalytic tetrad are highlighted within a red square. Species with SECIS elements are indicated by a red star. Numbers indicate bootstrap values from 2000 replicates. Brach lengths are drawn to scale as indicated by the scale bar. See Table 1 for ciliate species identification.

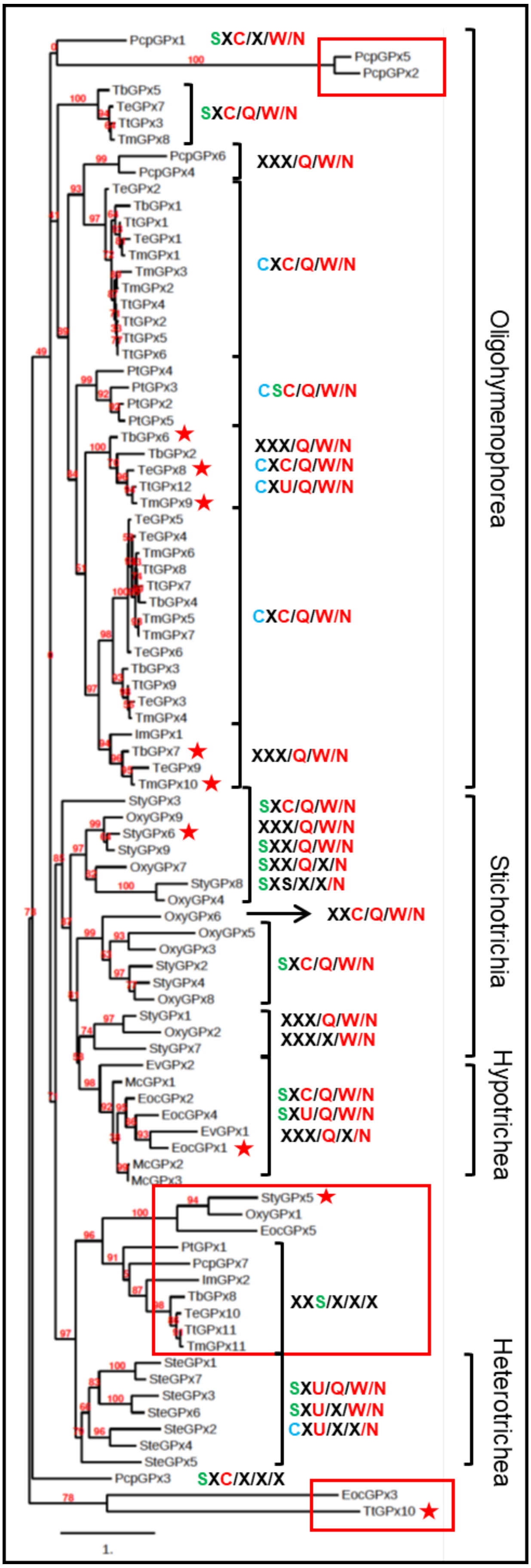
PcpGPx1
SXC/X/W/N
PcpGPx5
PcpGPx2
TbGPx5
TeGPx7
TtGPx3
TmGPx8
SXC/Q/W/N
PcpGPx6
PcpGPx4
XXX/Q/W/N
TeGPx2
TbGPx1
TtGPx1
TeGPx1
TmGPx1
TmGPx3
TmGPx2
TtGPx4
TtGPx2
TtGPx5
TtGPx6
CXC/Q/W/N
PtGPx4
PtGPx3
PtGPx2
PtGPx5
CSC/Q/W/N
TbGPx6
TbGPx2
TeGPx8
TtGPx12
TmGPx9
XXX/Q/W/N
CXC/Q/W/N
CXU/Q/W/N
TeGPx5
TeGPx4
TmGPx6
TtGPx8
TtGPx7
TbGPx4
TmGPx5
TmGPx7
TeGPx6
TbGPx3
TtGPx9
TeGPx3
TmGPx4
CXC/Q/W/N
ImGPx1
TbGPx7
TeGPx9
TmGPx10
XXX/Q/W/N
Oligohymenophorea
StyGPx3
OxyGPx9
StyGPx6
StyGPx9
OxyGPx7
StyGPx8
OxyGPx4
SXC/Q/W/N
XXX/Q/W/N
SXX/Q/W/N
SXX/Q/X/N
SXS/X/X/N
OxyGPx6
XXC/Q/W/N
OxyGPx5
OxyGPx3
StyGPx2
StyGPx4
OxyGPx8
SXC/Q/W/N
StyGPx1
OxyGPx2
StyGPx7
XXX/Q/W/N
XXX/X/W/N
Stichotrichia
EvGPx2
McGPx1
EocGPx2
EocGPx4
EvGPx1
EocGPx1
McGPx2
McGPx3
SXC/Q/W/N
SXU/Q/W/N
XXX/Q/X/N
Hypotrichea
StyGPx5
OxyGPx1
EocGPx5
PtGPx1
PcpGPx7
ImGPx2
TbGPx8
TeGPx10
TtGPx11
TmGPx11
XXS/X/X/X
SteGPx1
SteGPx7
SteGPx3
SteGPx6
SteGPx2
SteGPx4
SteGPx5
SXU/Q/W/N
SXU/X/W/N
CXU/X/X/N
Heterotrichea
PcpGPx3
SXC/X/X/X
EocGPx3
TtGPx10
1.
